# Methylation-dependent regulation of HIF-1α stability restricts retinal and tumour angiogenesis

**DOI:** 10.1038/ncomms10347

**Published:** 2016-01-13

**Authors:** Yunho Kim, Hye Jin Nam, Junyeop Lee, Do Young Park, Chan Kim, Young Suk Yu, Dongha Kim, Se Won Park, Jinhyuk Bhin, Daehee Hwang, Ho Lee, Gou Young Koh, Sung Hee Baek

**Affiliations:** 1 Creative Research Initiatives Center for Chromatin Dynamics, School of Biological Sciences, Seoul National University, Seoul 151-742, South Korea; 2 Graduate School of Medical Science and Engineering, Korea Advanced Institute of Science and Technology, Daejeon 305-701, South Korea; 3 Department of Ophthalmology, Asan Medical Center, University of Ulsan College of Medicine, Seoul 138-736, South Korea; 4 Department of Medical Oncology, CHA Bundang Medical Center, CHA University, Seongnam 13496, South Korea; 5 Department of New Biology and Center for Plant Aging Research, Institute for Basic Science, DGIST, Daegu 711-873, South Korea; 6 Graduate School of Cancer Science and Policy, Research Institute, National Cancer Center, Gyeonggi-do 10408, South Korea

## Abstract

Hypoxia-inducible factor-1α (HIF-1α) mediates hypoxic responses and regulates gene expression involved in angiogenesis, invasion and metabolism. Among the various HIF-1α posttranslational modifications, HIF-1α methylation and its physiological role have not yet been elucidated. Here we show that HIF-1α is methylated by SET7/9 methyltransferase, and that lysine-specific demethylase 1 reverses its methylation. The functional consequence of HIF-1α methylation is the modulation of HIF-1α stability primarily in the nucleus, independent of its proline hydroxylation, during long-term hypoxic and normoxic conditions. Knock-in mice bearing a methylation-defective *Hif1a*^*KA/KA*^ allele exhibit enhanced retinal angiogenesis and tumour vascularization via HIF-1α stabilization. Importantly, S28Y and R30Q mutations of HIF-1α, found in human cancers, are involved in the altered HIF-1α stability. Together, these results demonstrate a role for HIF-1α methylation in regulating protein stability, thereby modulating biological output including retinal and tumour angiogenesis, with therapeutic implications in human cancer.

## Abstract (web summary)

HIF-1α is a pivotal protein involved in angiogenesis and is known to be regulated posttranslationally. Here, the authors show that HIF-1α is methylated by Set7/9 methyltransferase, which reduces protein stability and contributes to reduced angiogenesis.

## Graphical abstract

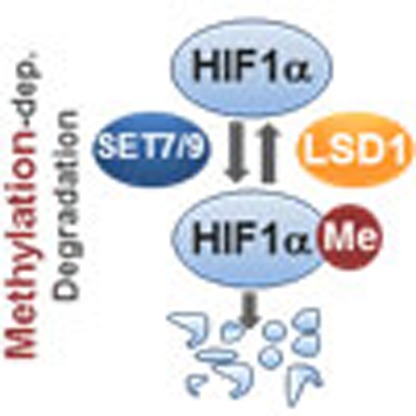

Hypoxia is a state in which the oxygen concentration is relatively lower than that of homeostasis under normoxic conditions^1^,^2^,^3^. Oxygen is one of the most significant elements for the metabolic regulation of the organism, because the lack of oxygen leads to improper energy production levels. In this state, cells reduce oxygen consumption to adapt to hypoxia and to maintain homeostasis. Hypoxia occurs under physiological and pathological conditions, such as ischaemia and wound healing, and in embryonic stem cell and solid tumour microenvironments^4^,^5^,^6^,^7^,^8^,^9^,^10^. Hypoxic responses are mediated by hypoxia-inducible factor-1 (HIF-1), a heterodimeric transcription factor that is composed of an oxygen-regulated α-subunit (HIF-1α or HIF-2α) and a constitutively expressed β-subunit (HIF-1β)^11^,^12^. HIF-1α is unstable under normoxic conditions, whereas HIF-1α is stabilized under hypoxic conditions. The HIF-1α/β heterodimer is recruited to a hypoxia response element and activates target gene expression involved in vascularization, glucose transport, energy metabolism and cell migration, to adapt to low oxygen conditions.

Regulating HIF-1α stability is an important step in adapting to hypoxic conditions. Under normoxic conditions, HIF-1α is hydroxylated by prolyl hydroxylase domain (PHD)-containing protein 1/2/3 and then the von Hippel–Lindau (VHL) tumour suppressor protein recognizes hydroxylated HIF-1α for degradation by the cullin2 E3 ligase complex^13^,^14^,^15^,^16^,^17^. In contrast, under hypoxic conditions, PHDs use oxygen as a cofactor and the enzymatic activities of PHDs decrease. Therefore, HIF-1α hydroxylation decreases, leading to HIF-1α stabilization. Not only hydroxylation but also other posttranslational modifications including SUMOylation, acetylation and phosphorylation are known to regulate HIF-1α functions. Previous studies have shown that HIF-1α is stabilized by SENP1, which desumoylates HIF-1α and inhibits the interaction between HIF-1α and VHL^18^. HIF-1α phosphorylation by p38 contributes to the inhibition of binding to VHL during ischaemia^19^. In contrast, HIF-1α acetylation has been shown to induce VHL-mediated ubiquitination of HIF-1α^20^.

HIF-1α plays a crucial role in physiological and pathophysiological angiogenesis by directly regulating vascular emdothelial growth factor (VEGF), a master regulator of angiogenesis in endothelial cells. *Hif1a*-null embryos die at E10.5 due to defective vessel formation in the placenta, yolk sac and branchial arches^21^,^22^. *Phd1/3* double knockout (KO) mice and *Phd2* conditional KO mice show erythemic appearances^23^. Abnormal HIF-1α regulation causes uncontrolled blood vessel growth and numerous vascular diseases^24^,^25^. In *Hif1a*^+/−^ mice, femoral artery ligation experiments show decreased limb perfusion and increased spontaneous amputation^5^, indicating that HIF-1α plays a role in blood flow during hindlimb ischaemia. HIF-1α gain-of-function in mice increases VEGF expression, microvessel density, tumour growth and angiogenesis, whereas HIF-1α loss-of-function in mice inhibits tumour growth and angiogenesis^26^,^27^.

In recent times, several reports have indicated that protein methylation can be recognized as a modification that regulates protein stability^28^,^29^. We reported that methylation-specific ubiquitination machinery includes the damage-specific DNA-binding protein 1 (DDB1)/cullin 4 (CUL4) E3 ubiquitin ligase complex and a DDB1–CUL4-associated factor 1 adaptor, which recognizes monomethylated substrates induced by enhancer of zeste homologue 2 (EZH2) methyltransferase^30^. SET7/9 is a SET domain-containing methyltransferase that acts on histone H3K4 and on several non-histone proteins including DNA methyltransferase 1 (DNMT1), E2F1 and signal transducer and activator of transcription 3 (refs 31, 32, 33). SET7/9-dependent methylation of DNMT1 and E2F1 regulates the stability of these proteins^32^,^34^. Lysine-specific demethylase 1 (LSD1, also known as AOF2 or BHC110) demethylates mono- and dimethylated H3K4 or H3K9 via an amine oxidase reaction^35^,^36^. LSD1 interacts with androgen receptor *in vitro* and *in vivo*, and stimulates androgen receptor-dependent transcription^35^. LSD1 may switch the substrate H3K4me1/2 to H3K9me1/2 in the context of androgen receptor gene regulation. In addition to its role as a histone demethylase, LSD1 demethylates non-histone proteins such as p53 and Dnmt1. LSD1 controls the tumour suppressor activity of p53 via demethylation^37^. LSD1 plays essential roles in maintaining global methylation in embryonic stem cells by regulating Dnmt1 demethylation^31^.

In this study, we provide evidence that LSD1-mediated demethylation of HIF-1α leads to HIF-1α stabilization under hypoxic conditions. To validate the *in vivo* function of HIF-1α methylation, we generate a methylation-deficient *Hif1a*^*KA/KA*^ knock-in mouse and characterize the phenotypes of enhanced retinal angiogenesis and tumour growth and angiogenesis promotion via HIF-1α stabilization. Furthermore, we discuss the physiological relevance of HIF-1α methylation-dependent regulation of protein stability in human cancers.

## Results

### SET7/9-mediated HIF-1α methylation occurs in the nucleus

**Figure 1:**
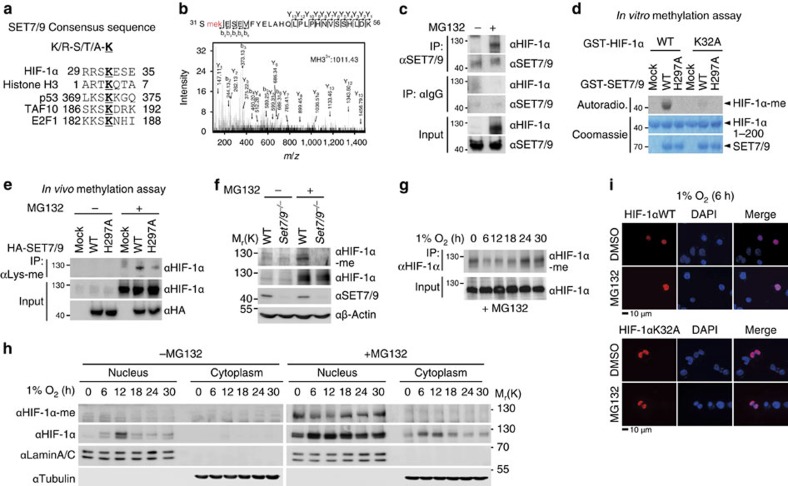
Identification of HIF-1α methylation by SET7/9 methyltransferase at the K32 residue. (**a**) Identification of a putative SET7/9 methylation site in HIF-1α. (**b**) Mass spectrometric analysis of HIF-1α purified from HeLa cells indicates HIF-1α methylation at the K32 residue. (**c**) Co-immunoprecipitation of endogenous HIF-1α with SET7/9 from HeLa cells treated with or without MG132. (**d**) *In vitro* methylation assay of HIF-1α WT or K32A proteins was performed with either purified SET7/9 WT or enzymatic MT (H297A) proteins. (**e**) HIF-1α methylation was determined in HeLa cells expressing either SET9 WT or H297A MT. Immunoprecipitation assay with anti-methyl lysine antibody, followed by immunoblot (IB) analysis with anti-HIF-1α antibody was performed. (**f**) HIF-1α methylation level was determined in WT or *Set7/9*^*−/−*^ MEFs treated with or without MG132. (**g**) Immunoprecipitation with anti-HIF-1α antibody from HeLa cells treated with MG132, followed by IB analysis with anti-HIF-1α-me antibody. (**h**) Nuclear and cytoplasmic fractionation of HeLa cells was performed and methylated HIF-1α levels were monitored. HeLa cells were exposed to hypoxic conditions with or without MG132 for the indicated times. Lamin A/C was used as a nuclear marker and tubulin was used as a cytoplasmic marker. (**i**) HeLa cells were transfected with Flag-HIF-1α WT or K32A MT in the presence or absence of MG132 under hypoxic condition. Cells were stained with anti-Flag antibody (red) as indicated. The nuclei were stained with 4,6-diamidino-2-phenylindole (DAPI, blue). Scale bar, 10 μm.

Although ubiquitination, SUMOylation, acetylation and proline hydroxylation of HIF-1α have been reported to play important roles in regulating HIF-1α functions^38^, physiological roles of HIF-1α methylation have not yet been elucidated. As protein methylation is conducted by protein methyltransferases, we examined whether HIF-1α possesses a consensus sequence targeted by specific methyltransferases. Near the lysine 32 site of HIF-1α, we found a SET7/9-specific recognition motif designated by [K/R]-[S/T/A]-K (in which the methylation lysine site is underlined; Fig. 1a)^39^,^40^,^41^. We performed liquid chromatography mass spectrometry/mass spectrometry (LC-MS/MS) analysis for HIF-1α and confirmed that HIF-1α is methylated at the lysine 32 residue (Fig. 1b)^41^. The association of SET7/9 with HIF-1α was validated by co-immunoprecipitation assays at endogenous expression levels in the absence or presence of MG132 (Fig. 1c). As endogenous HIF-1α protein level under normoxic condition is detectable only in the presence of MG132 (ref. 42), we found the association of SET7/9 with HIF-1α in the presence of MG132.

We performed an *in vitro* methylation assay using purified GST-SET7/9 proteins as enzymes and GST-HIF-1α proteins as substrates. The introduction of SET7/9 increased HIF-1α methylation; however, the mutagenesis of lysine to alanine almost completely abolished HIF-1α methylation at the K32 site, as assessed by an *in vitro* methylation assay (Fig. 1d). To determine whether the catalytic activity of SET7/9 is required for HIF-1α methylation, either SET7/9 wild-type (WT) or an H297A mutant (MT) with impaired methyltransferase activity was introduced^43^. Immunoprecipitation assay with anti-methyl-lysine antibodies revealed that the SET7/9 WT, but not H297A MT, significantly induced HIF-1α methylation (Fig. 1e), indicating that SET7/9 is responsible for HIF-1α methylation in an enzymatic activity-dependent manner. A specific antibody for methylated HIF-1α at K32 was generated using a methylated HIF-1α peptide and dot blot analysis confirmed that this antibody recognized the methylated peptide specifically (Supplementary Fig. 1a). *Set7/9*-deficient primary mouse embryonic fibroblasts (MEFs) exhibited significantly reduced HIF-1α methylation level compared with WT MEFs and methylated HIF-1α was detected only from WT MEFs in the presence of MG132 (Fig. 1f).

Next, we determined when and where HIF-1α methylation occurs. HIF-1α methylation was detected under normoxic conditions with MG132 treatment, to inhibit 26S proteasome-dependent degradation, and hypoxic challenge led to decreased HIF-1α methylation (Fig. 1g). Intriguingly, this reduced HIF-1α methylation level under hypoxic conditions was restored during long-term hypoxia (Fig. 1g). Using the anti-methyl HIF-1α antibody, we further examined whether HIF-1α methylation occurs in the cytoplasm or the nucleus and found that HIF-1α methylation occurred primarily in the nucleus (Fig. 1h). We examined the potential effects of methylation on HIF-1α localization and immunostaining data revealed that both HIF-1α WT and K32A MT, which is deficient of methylation, remained exclusively in the nucleus in the absence or presence of MG132 in hypoxic condition (Fig. 1i). These data indicate that SET7/9-mediated HIF-1α methylation, which occurs in the nucleus, does not affect the subcellular localization of HIF-1α.

### HIF-1α demethylation by LSD1 increases HIF-1α stability

**Figure 2:**
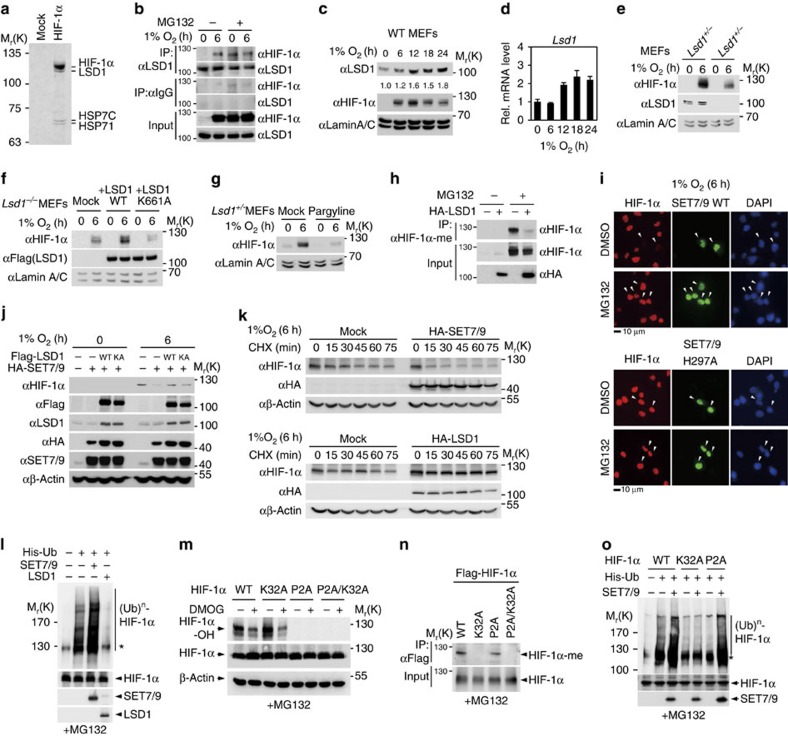
LSD1-mediated HIF-1α demethylation increases HIF-1α protein stability. (**a**) HIF-1α-interacting proteins were purified from HEK293T cells under hypoxic conditions by Flag-M2 agarose. Bound proteins were resolved by SDS–PAGE and prepared for LC-MS/MS analysis. (**b**) Co-immunoprecipitation of endogenous LSD1 with HIF-1α from HeLa cells in the absence or presence of MG132. LSD1 and HIF-1α protein levels of nuclear fraction in WT MEFs (**c**) and LSD1 mRNA levels (**d**) were monitored under hypoxic conditions for the indicated times. Values are expressed as mean±s.d. (*n*=3). (**e**) HIF-1α protein levels of nuclear fraction in *Lsd1*^*+/−*^ and *Lsd1*^*−/−*^ MEFs were compared in the presence or absence of hypoxic challenge for 6 h. (**f**) *Lsd1*^*−/−*^ MEFs were reconstituted with either WT or a catalytically inactive MT (K661A) of LSD1. HIF-1α protein levels of nuclear fraction were monitored. (**g**) Nuclear HIF-1α protein levels were compared. *Lsd1*^*+/−*^ MEFs were pretreated with pargyline for 12 h and exposed to hypoxic conditions for 6 h. (**h**) HIF-1α methylation was decreased in HeLa cells by overexpressing LSD1 with MG132 treatment. (**i**) IF assay was performed in HeLa cells with the indicated antibodies. Cells were exposed to hypoxic condition for 6 h with or without MG132 treatment. Scale bar, 10 μm. (**j**) IB analysis of HeLa cells expressing the indicated proteins was performed. (**k**) HeLa cells expressing indicated proteins were incubated in a hypoxia chamber for 6 h and treated with CHX (20 μg ml^−1^), collected at the indicated times and analysed by IB assay. (**l**) Protein extracts from HeLa cells co-transfected with the indicated plasmids were subjected to pull-down with Ni^2+^-NTA beads. HIF-1α ubiquitination was assessed by anti-HIF-1α antibody in the presence of MG132. (**m**) HIF-1α hydroxylation was determined in HeLa cells expressing HIF-1α WT, K32A, P2A or P2A/K32A MT with or without DMOG in the presence of MG132. (**n**) Immunoprecipitation with anti-Flag antibody from HeLa cells expressing Flag-HIF-1α WT, K32A, P2A or P2A/K32A MT in the presence of MG132, followed by IB analysis with anti-HIF-1α-me antibody was performed. (**o**) SET7/9-dependent HIF-1α ubiquitination was determined after transfection with HIF-1α WT, K32A or P2A MT. Ubiquitination assay was performed as in **l**.

To understand the function of HIF-1α methylation, we identified HIF-1α-interacting proteins involved specifically in protein methylation and demethylation processes by affinity chromatography. Intriguingly, LSD1 histone demethylase was identified as a HIF-1α-interacting protein from LC-MS/MS analysis (Fig. 2a and Supplementary Data 1). Co-immunoprecipitation assay confirmed that HIF-1α and LSD1 bound at endogenous expression levels under hypoxic condition or in the presence of MG132 (Fig. 2b). LSD1 protein level was induced on hypoxia in WT MEFs (Fig. 2c). As *LSD1* has been shown to be a hypoxia target gene^44^, we measured messenger RNA level of LSD1 on hypoxia and found the slight induction of LSD1 mRNA level on hypoxia (Fig. 2d).

We found that the protein levels of HIF-1α in *Lsd1*^−/−^ MEFs were decreased significantly compared with those in *Lsd1*^+/−^ MEFs (Fig. 2e). To further examine whether LSD1 enzymatic activity is required for regulating HIF-1α protein stability, reconstitution experiments with either LSD1 WT or an enzymatically inactive LSD1 K661A MT in *Lsd1*^−/−^ MEFs were performed. Increased HIF-1α protein levels were restored in only LSD1 WT-reconstituted cells but not in LSD1 K661A MT-reconstituted cells under hypoxic conditions (Fig. 2f). Treatment of pargyline, an LSD1 inhibitor, which blocks LSD1 enzymatic activity, led to the HIF-1α destabilization (Fig. 2g). As HIF-1α is methylated by SET7/9, we examined whether LSD1 is responsible for HIF-1α demethylation. Indeed, LSD1 led to HIF-1α demethylation both *in vivo* and *in vitro* (Fig. 2h and Supplementary Fig. 1b). However, enzymatic activity of LSD1 did not affect binding affinity to HIF-1α (Supplementary Fig. 1c) and the amino-terminal domain of HIF-1α encompassing 1–200 amino acids showed direct binding to LSD1 (Supplementary Fig. 1d). Co-immunoprecipitation assay revealed that HIF-1α and LSD1 showed comparable binding in the absence or presence of SET7/9 (Supplementary Fig. 1e), confirming that the binding between HIF-1α and LSD1 was not affected by HIF-1α methylation status. As expected, *Hif-1a* target gene activation on hypoxia was further attenuated in *Lsd1*^−*/*−^ MEFs, but it was further activated in *Set7/9*^−/−^ MEFs (Supplementary Fig. 1f,g).

To further examine whether the methylation status of HIF-1α affects its protein stability, we performed immunofluorescence (IF) assay in the absence or presence of MG132 under hypoxic conditions. The introduction of SET7/9 WT reduced HIF-1α protein levels under hypoxic conditions, whereas the SET7/9 enzymatic MT failed to affect HIF-1α stability (Fig. 2i). MG132 treatment blocked the SET7/9-dependent decrease in HIF-1α protein levels under hypoxic conditions. Next, we examined whether the introduction of LSD1 antagonizes the SET7/9-dependent destabilization of HIF-1α proteins. IB analysis showed that the introduction of SET7/9 reduced HIF-1α protein levels, and that LSD1 WT, but not the LSD1 enzymatic MT, blocked the SET7/9-dependent decrease in endogenous HIF-1α protein levels on hypoxia (Fig. 2j). Treatment of the protein synthesis inhibitor, cycloheximide showed that SET7/9 overexpression significantly decreased the half-life of endogenous HIF-1α, whereas LSD1 overexpression increased the half-life of HIF-1α (Fig. 2k). To further examine whether the 26S proteasome-dependent degradation pathway is involved in the regulation of HIF-1α protein levels, we performed HIF-1α ubiquitination assay with SET7/9 or LSD1 in the presence of MG132. Indeed, SET7/9 significantly increased HIF-1α ubiquitination and the introduction of LSD1 almost completely abolished the increase in HIF-1α ubiquitination (Fig. 2l).

HIF-1α is hydroxylated by PHD1/2/3 in the cytosol and the hydroxylated HIF-1α is subject to degradation by the CUL2 E3 ubiquitin ligase complex under normoxic conditions. Under hypoxic conditions, the enzymatic activities of PHDs are decreased and the decrease in HIF-1α hydroxylation leads to HIF-1α stabilization^13^,^15^. Therefore, we examined whether HIF-1α methylation affects its hydroxylation. A HIF-1α methylation-deficient K32A MT showed comparable hydroxylation levels and treating this MT-expressing cells with dimethyloxalylglycine (DMOG), a prolyl hydroxylase inhibitor, attenuated HIF-1α hydroxylation as in the case of HIF-1α WT (Fig. 2m), indicating that HIF-1α methylation is independent of its hydroxylation. In parallel, a HIF-1α hydroxylation-deficient P2A MT (P402A/P564A) exhibited methylation levels comparable to those of WT, indicating that HIF-1α hydroxylation does not affect SET7/9-dependent methylation in the nucleus (Fig. 2n). We further examined the ubiquitination of HIF-1α WT, K32A MT or P2A MT in the presence of MG132 and found that HIF-1α K32A mutation led to a marked reduction in HIF-1α ubiquitination in the presence of SET7/9, in contrast to HIF-1α WT and P2A MT (Fig. 2o). The methylated HIF-1α proteins are found to be hydroxylated as well (Supplementary Fig. 1h,i). Together, these data indicate that LSD1-dependent demethylation of HIF-1α stabilizes HIF-1α proteins under hypoxic conditions by reversing SET7/9-mediated HIF-1α methylation-dependent degradation by 26S proteasomes, which is independent of HIF-1α hydroxylation.

### *Hif1a* ^*KA/KA*^ knock-in mice display a haematologic abnormality

**Figure 3:**
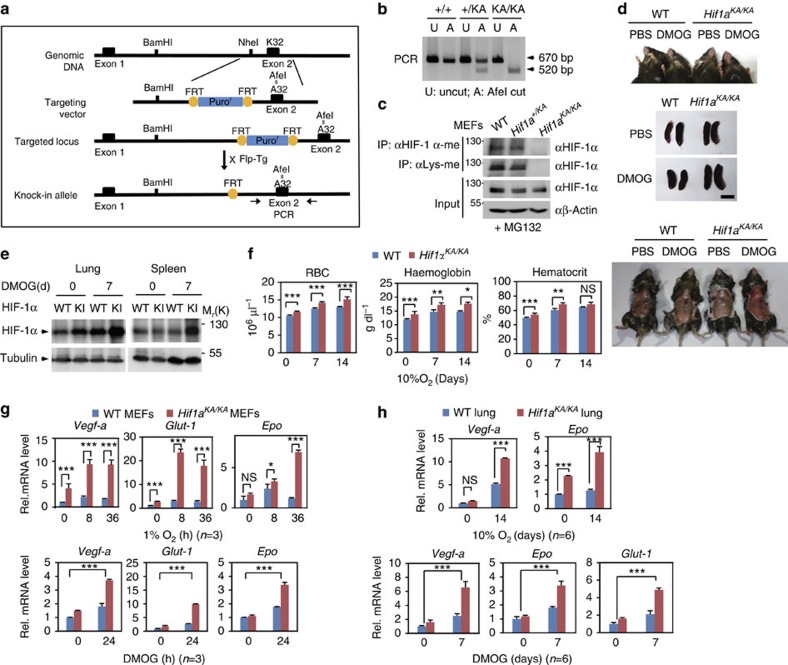
*Hif1a*^*KA/KA*^ knock-in mice show a haematologic abnormality with elevated Epo levels. (**a**) Strategy for the generation of *Hif1a*^*KA/KA*^ knock-in mice. Site-directed mutagenesis introduced lysine to alanine substitutions, which generated the AfeI site. (**b**) Genotyping of WT, *Hif1a*^*+/KA*^ and *Hif1a*^*KA/KA*^ mice. The *Hif1a*^*KA/KA*^ allele but not the WT allele was digested by the AfeI restriction enzyme. A, AfeI cut; U, uncut. (**c**) HIF-1α methylation levels were assessed in WT, *Hif1a*^*+/KA*^ and *Hif1a*^*KA/KA*^ MEFs. (**d**) Phenotypes of WT and *Hif1a*^*KA/KA*^ mice treated with DMOG or PBS for 7 days. Upper panel: paws and snouts; middle panel: spleens. Scale bar, 10 mm; lower panel: peritonea. (**e**) IB analysis was performed using anti-HIF-1α antibody to monitor HIF-1α protein levels in mouse lung and spleen extracts. Mice were treated with DMOG or PBS for 7 days. (**f**) Haematological parameters of peripheral blood from WT and *Hif1a*^*KA/KA*^ mice. Hb, haemoglobin; Hct, haematocrit; RBC, red blood cells. Data are expressed as mean±s.d. (WT mice: *n*=7 in normoxic condition, *n*=6 in hypoxic condition for 7 days, *n*=4 in hypoxic condition for 14 days; *Hif1a*^*KA/KA*^ mice: *n*=7 in normoxic condition, *n*=5 in hypoxic condition for 7 days, *n*=4 in hypoxic condition for 14 days, one-way analysis of variance (ANOVA), *P<0.05, **P<0.01, ***P<0.001). (**g**) mRNA levels of *Vegf-a*, *Glut-1* and *Epo* in WT and *Hif1a*^*KA/KA*^ MEFs were compared at the indicated times of hypoxic conditions (upper panel) or DMOG treatment (lower panel). Data are expressed as mean±s.d. (*n*=3 for each group, one-way ANOVA or two-way ANOVA, *P<0.05, **P<0.01, ***P<0.001). (**h**) mRNA levels of *Vegf-a* and *Epo* in WT and *Hif1a*^*KA/KA*^ mouse lung extracts were compared. Mice were incubated in a hypoxia chamber for 14 days (upper panel) or treated with DMOG for 7 days (lower panel). Data are expressed as mean±s.d. (*n*=6 for each group, one-way ANOVA or two-way ANOVA,*P<0.05, **P<0.01, ***P<0.001).

To examine the roles of HIF-1α methylation *in vivo*, we generated *Hif1a*^*KA/KA*^ knock-in mice. To replace lysine 32 of HIF-1α with alanine in the mouse genome, we designed a knock-in MT targeting vector, which contained substituted DNA sequences in the second exon and an flippase recognition target (FRT)-flanked puromycin-resistant (Puro^r^) cassette in intron. Lysine to alanine substitution was introduced by site-directed mutagenesis, which generated the *Afe*I site (Fig. 3a). The knock-in of *Hif1a*^*KA/KA*^ was confirmed by both *Afe*I digestion and PCR product sequencing using allele-specific primers (Fig. 3b). *Hif1a*^*KA/KA*^ mice have been backcrossed to a C57BL/6 background for at least seven generations. No lethality was associated with the targeted *Hif1a*^*KA/KA*^ alleles, as *Hif1a*^*KA/KA*^ mice were normal from birth until adulthood and largely indistinguishable from their WT or heterozygote littermates in viability and fertility, exhibiting an expected Mendelian distribution ratio. First, we determined whether HIF-1α methylation was abolished in primary MEFs obtained from *Hif1a*^*KA/KA*^ mice compared with those from heterozygous and WT mice. Immunoprecipitation assay with anti-HIF-1α-me antibodies confirmed that HIF-1α methylation was not detected in *Hif1a*^*KA/KA*^ MEFs in contrast to WT and heterozygous MEFs (Fig. 3c). Compared with HIF-1α protein level, HIF-2α protein level was comparable in WT, *Hif1a*^*+/KA*^ and *Hif1a*^*KA/KA*^ mice (Supplementary Fig. 2a,b).

Elevated HIF-1α levels are associated with increased erythropoietin (Epo) levels, leading to erythrocytosis^45^,^46^,^47^. To examine the possibility that a lack of HIF-1α methylation leads to erythrocytosis via increased HIF-1α protein levels, we examined the blood phenotype in *Hif1a*^*KA/KA*^ mice compared with that in WT mice. WT and *Hif1a*^*KA/KA*^ mice were injected with DMOG to eliminate hydroxylation effect and the phenotypes were monitored. *Hif1a*^*KA/KA*^ mice exhibited reddened snouts, paws and peritoneum along with enlarged spleens (Fig. 3d). HIF-1α protein level was increased in the lungs and spleens of *Hif1a*^*KA/KA*^ mice treated with DMOG compared with WT mice (Fig. 3e). We also examined the haematological parameters of peripheral blood from *Hif1a*^*KA/KA*^ mice compared with WT mice. *Hif1a*^*KA/KA*^ mice had significantly increased numbers of red blood cells along with high haemoglobin concentrations (Fig. 3f and Supplementary Fig. 2c). Haematocrit values were also increased in *Hif1a*^*KA/KA*^ mice compared with WT mice (Fig. 3f and Supplementary Fig. 2c). Furthermore, *Vegf-a*, *Glut-1* and *Epo* mRNA levels were increased in *Hif1a*^*KA/KA*^ MEFs compared with WT MEFs on exposure to hypoxic conditions or DMOG treatment for 24 h (Fig. 3g). *Vegf-a* and *Epo* mRNA levels were increased in *Hif1a*^*KA/KA*^ lung extracts compared with WT on exposure to hypoxic conditions for 14 days or DMOG treatment for 7 days (Fig. 3h). IB analysis revealed that protein level of EPO is also increased in *Hif1a*^*KA/KA*^ lung extracts compared with WT (Supplementary Fig. 2d). Together, these data indicate that *Hif1a*^*KA/KA*^ mice had haematologic abnormalities and enhanced HIF-1α levels.

### Increased cell motility and tumour growth in *Hif1a* ^*KA/KA*^ MEFs

**Figure 4:**
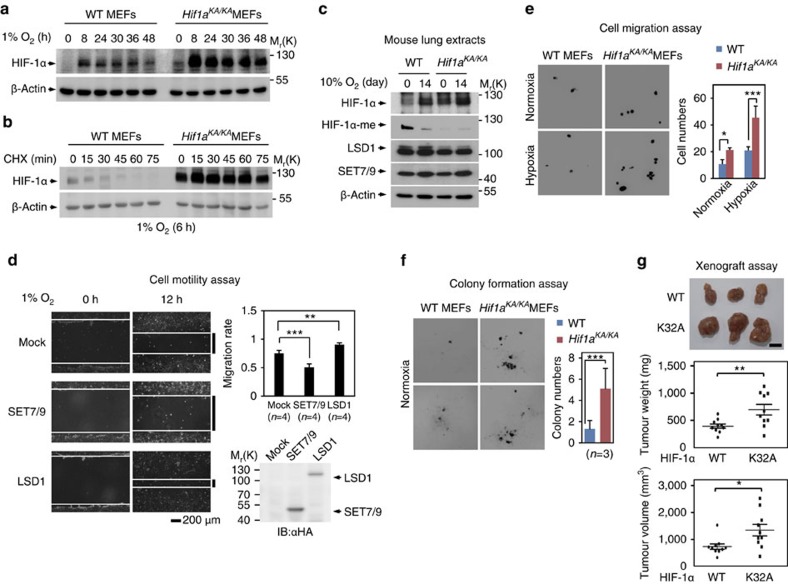
Altered cell function and tumour growth of *Hif1a*^*KA/KA*^ MEFs. (**a**) HIF-1α protein levels in WT and *Hif1a*^*KA/KA*^ MEFs were compared at the indicated times after hypoxic challenge. (**b**) The half-lives of HIF-1α in WT and *Hif1a*^*KA/KA*^ MEFs were compared in cells treated with cycloheximide (CHX) at the indicated times after 6 h of hypoxic challenge. (**c**) IB analysis of mouse lung extracts was performed. Mice were incubated in a hypoxia chamber (10% O_2_) for 14 days. (**d**) Representative photomicrographs from a scratch-cell motility assay of MEFs expressing either SET7/9 or LSD1 under hypoxic conditions for the indicated times. Migration rate was calculated and expressed as ratio of cell coverage to the initial cell-free zone. Scale bar, 200 μm. Data are expressed as mean±s.d. for four independent experiments (*n*=4 for each group, *t*-test, **P<0.01, ***P<0.001). (**e**) Photomicrographs from Transwell cell migration assays of WT and *Hif1a*^*KA/KA*^ MEFs under normoxic and hypoxic conditions for 12 h. Representative images are shown for each group. Bar graph shows the mean number of cells per filter±s.d. (*n*=4 for each group, *t*-test, ***P<0.001). (**f**) Anchorage-independent growth of WT and *Hif1a*^*KA/KA*^ primary MEFs in soft agar. Representative images are shown for each group. Values are expressed as mean±s.d. (*n*=3, *t*-test, ***P<0.001). Colonies were counted in 15 fields. (**g**) *In vivo* xenograft assay of MDA-MB231 cells stably expressing HIF-1α shRNA with reconstituted HIF-1α WT or K32A. Vertically presented tumours were derived from same nude mouse at the left flank, WT at the right flank, K32A. Scale bar, 10 mm. Tumours were excised from nude mice and tumour weight and volumes were measured (*n*=10, *t*-test, *P<0.05, **P<0.01).

HIF-1α expression was drastically enhanced in *Hif1a*^*KA/KA*^ MEFs compared with WT MEFs on exposure to hypoxic conditions at the indicated time points (Fig. 4a). We monitored the half-life of HIF-1α in WT and *Hif1a*^*KA/KA*^ MEFs and found that the half-life of HIF-1α in *Hif1a*^*KA/KA*^ MEFs treated with cycloheximide was further extended (Fig. 4b). We also compared the HIF-1α protein levels along with its methylation level from mouse lung extracts after incubating WT and *Hif1a*^*KA/KA*^ mice in hypoxic chambers for 14 days. HIF-1α protein levels were increased concomitant with decreased HIF-1α methylation levels on exposure to hypoxic conditions in mouse lung extracts (Fig. 4c).

To examine whether SET7/9 and LSD1 modulate cell motility, we performed cell motility assays by overexpressing SET7/9 or LSD1 in MEFs in the absence or presence of hypoxic challenge. The introduction of SET7/9 resulted in decreased cell motility, whereas the introduction of LSD1 increased cell motility on exposure to hypoxic conditions (Fig. 4d). As increased HIF-1α levels are related to cancerous phenotypes under hypoxic conditions, we examined whether *Hif1a*^*KA/KA*^ MEFs exhibit a greater number of cancerous phenotypes than WT MEFs by performing cell migration and colony formation assays. Compared with WT MEFs, *Hif1a*^*KA/KA*^ MEFs showed increased cell migration (Fig. 4e). Furthermore, colony formation assay revealed that *Hif1a*^*KA/KA*^ MEFs showed increased colony numbers compared with WT (Fig. 4f). To examine whether HIF-1α methylation could negatively regulate tumorigenic behaviour *in vivo*, we injected MDA-MB231 cells stably expressing HIF-1α WT and K32A MT subcutaneously into athymic nude mice. HIF-1α K32A MT-expressing cells resulted in the increased tumour formation, weight and volume compared with the cells expressing HIF-1α WT (Fig. 4g, Supplementary Fig. 2e,f). Hence, ectopically expressing methylation-defective HIF-1α K32A MT provides cells with tumour growth advantage.

### Enhanced retinal angiogenesis in *Hif1a* ^*KA/KA*^ knock-in mice

**Figure 5:**
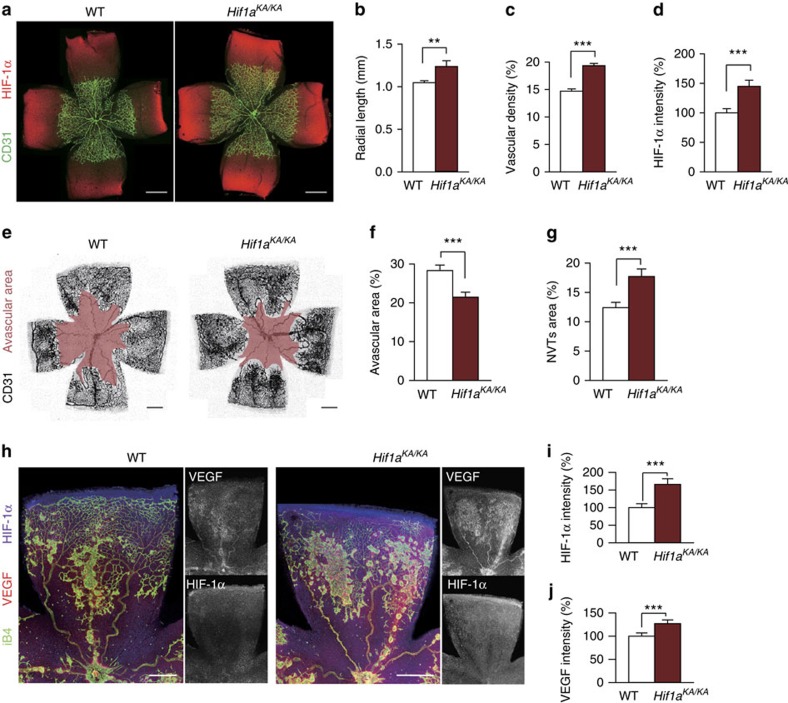
Enhanced retinal angiogenesis in *Hif1a*^*KA/KA*^ knock-in mice. (**a**–**d**) Physiological retinal angiogenesis at postnatal day 5 (P5) in WT and *Hif1a*^*KA/KA*^ mice. (**a**) P5 retina whole mount-stained with anti-CD31 and anti-HIF-1α antibodies. Scale bars, 500 μm. (**b**–**d**) Comparisons of blood vessel radial length, vascular density and signal intensity of HIF-1α. Values are mean±s.d. (*n*=4 for each group, *t*-test, **P<0.01, *** *P*<0.001). (**e**–**j**) Pathologic retinal angiogenesis in an OIR model of WT and *Hif1a*^*KA/KA*^ mice. (**e**) P17 OIR retina whole mount stained with anti-CD31 antibody. Avascular areas are highlighted in pink. Scale bars, 500 μm. (**f**,**g**) Comparisons of avascular and neovascular tuft (NVT) areas. Values are mean±s.d. (*n*=4 for WT, *n*=8 for *Hif1a*^*KA/KA*^, *t*-test, ***P<0.001). (**h**) P17 OIR retina stained with anti-isolectin B4 (iB4), anti-VEGF and anti-HIF-1α antibodies. Scale bars, 500 μm. (**i**,**j**) Comparisons of signal intensities of HIF-1α and VEGF. Values are mean±s.d. (*n*=4 for each group, *t*-test, ***P<0.001).

Hypoxia-induced HIF-1α stabilization activates the transcription of several target genes encoding angiogenic growth factors, which stimulate the proliferation and migration of endothelial cells, leading to angiogenesis under both physiological and pathological conditions^48^. To investigate the role of HIF-1α methylation in physiologic angiogenesis, we examined retinal vascular growth during development at postnatal day 5 (P5) in WT, *Hif1a*^*KA/+*^ and *Hif1a*^*KA/KA*^ mice. WT mice and *Hif1a*^*KA/+*^ showed no significant difference in vascular phenotype in terms of radial length and vascular density (Supplementary Fig. 3a–c). However, compared with control WT mice, the retinal vessels of *Hif1a*^*KA/KA*^ mice displayed increased radial length (1.2-fold) and vascular density (1.3-fold; Fig. 5a–c). Protein levels of HIF-1α in ganglion cell layer were increased by 45% in hypoxic avascular area of the retina in *Hif1a*^*KA/KA*^ mice compared with control WT mice (Fig. 5a,d and Supplementary Fig. 3d). To examine the role of HIF-1α methylation in pathological angiogenesis, we generated an oxygen-induced retinopathy (OIR) model^49^ that mimics human ischaemic retinopathies. Compared with control WT mice, *Hif1a*^*KA/KA*^ mice exhibited reduced avascular areas of the retina (76%) but increased neovascular tuft areas (43%) (Fig. 5e–g), whereas *Hif1a*^*KA/+*^ showed no significant difference (Supplementary Fig. 3e–g). The retina of *Hif1α*^*KA/KA*^ mice showed higher HIF-1α (65%) and VEGF (30%) expression levels than those of control WT mice (Fig. 5h–j). These data indicate that HIF-1α stabilization in *Hif1a*^*KA/KA*^ mice accelerates compensatory vascular growth into the avascular retina and abnormal vascular growth under ischaemic conditions. Together, these findings indicate that deficiency in HIF-1α methylation leading to the HIF-1α stabilization enhances physiological or pathological angiogenesis.

### Increased tumour growth and angiogenesis in *Hif1a* ^*KA/KA*^ mice

**Figure 6:**
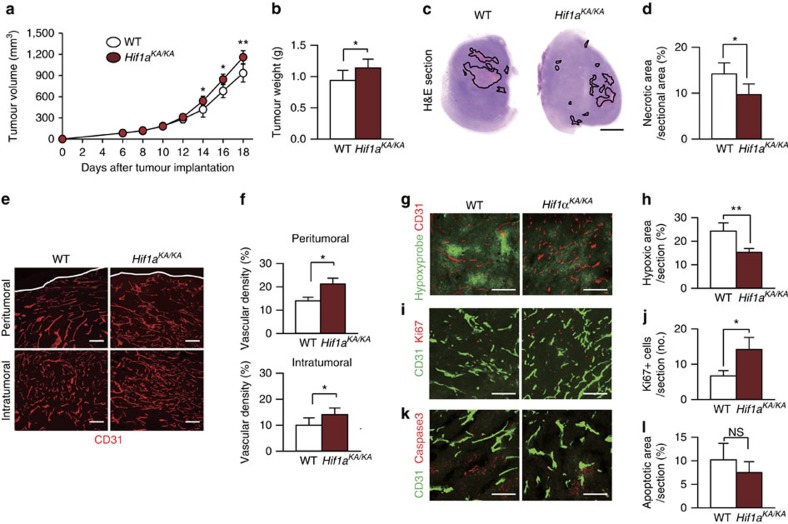
Mutation at the K32 site of HIF-1α promotes tumour growth and angiogenesis. (**a**–**l**) At 18 days after tumour cell implantation, tumour samples were harvested and histological analyses were performed. Unless otherwise indicated: scale bars, 200 μm. Values are mean±s.d. (*n*=14 for each group, *t*-test, *P<0.05, **P<0.01). (**a**) Comparison of tumour growth curves between WT and *Hif1a*^*KA/KA*^ mice after tumour implantation. (**b**) Comparison of tumour weights at the time of killing. (**c**) Tumour sections stained with haematoxylin and eosin (H&E). Black lines indicate intratumoral necrotic regions. Scale bar, 2 mm. (**d**) Comparison of intratumoral necrosis. The necrotic area is quantified as a percentage per total sectional area. (**e**,**f**) Images and comparison of CD31^+^ tumour blood vessels (red) in the peri- and intratumoral areas. White lines indicate the boundary of tumour. Scale bars, 200 μm. (**g**,**h**) Images and comparison of hypoxic area (green) in tumour centre. (**i**,**j**) Images and comparison of Ki67^+^ proliferating cells (red) in tumour centre. (**k**,**l**) Images and comparison of caspase3^+^ apoptotic cells (red) in tumour centre.

To determine the effects of HIF-1α methylation on tumour angiogenesis and progression, we employed a Lewis lung carcinoma (LLC) tumour model by subcutaneously implanting LLC tumour cells into the flanks of WT and *Hif1a*^*KA/KA*^ mice. At 18 days after tumour cell implantation, *Hif1a*^*KA/KA*^ mice displayed 24.0% and 26.1% increases in tumour volume and weight, respectively, compared with WT mice (Fig. 6a,b). In addition, intratumoural necrosis was 31.7% less in *Hif1a*^*KA/KA*^ mice compared with that in WT mice (Fig. 6c,d). Moreover, tumour vascular densities in the peritumoural and intratumoural areas were 47.2% and 44.1% higher, respectively, in *Hif1a*^*KA/KA*^ mice than those in WT mice (Fig. 6e,f), indicating that tumour angiogenesis was highly promoted in *Hif1a*^*KA/KA*^ mice. This enhanced tumour neovascularization attenuated intratumoural hypoxia in tumours of *Hif1a*^*KA/KA*^ mice compared with those of WT mice (Fig. 6g,h). Detailed analysis of tumour microenvironment also revealed 2.1-fold increased proliferation of tumour cells (Fig. 6i,j) with no remarkable difference in apoptosis in the centre of tumours of *Hif1a*^*KA/KA*^ mice compared with those of WT mice (Fig. 6k,l). Taken together, these findings indicate that HIF-1α methylation deficiency promotes tumour angiogenesis, thereby accelerating tumour growth.

### Biological relevance of HIF-1α methylation in human cancers

**Figure 7:**
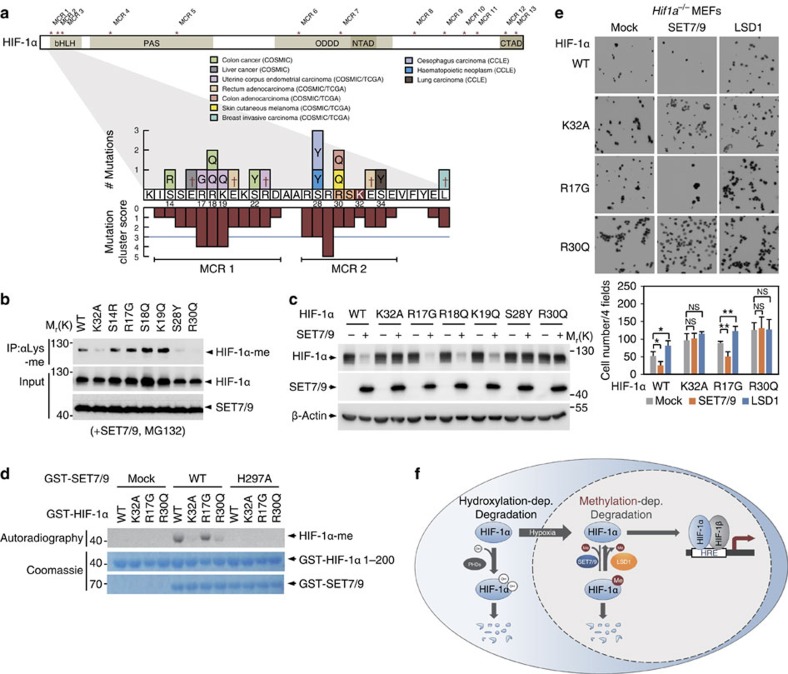
Biological significance of HIF-1α methylation in human cancers. (**a**) Schematics indicating known HIF-1α mutations in various cancer patients and cancer cell lines. For each amino acid, the mutation cluster score was calculated as the sum of the numbers of mutations of the amino acid and two neighbouring amino acids based on the scan statistics as previously described^68^. Mutation cluster regions (MCRs) were then determined as the regions within which the maximum mutation cluster score ⩾3 (random permutation test, *P*-value<0.05) and the number of amino acids ⩾5 and were denoted by asterisks in the entire protein structure of HIF-1α. MCRs 1 and 2 close to K32 were shown in detail with mutation cluster scores within the regions. Reference sequences for 12 to 40th amino acid residues of HIF-1α are denoted in the boxes on *x* axis. Sequence changes resulted from the mutations are shown in the boxes above the reference sequences and synonymous mutations are denoted by the cross (†) symbols. Numbers under the boxes indicate the presence of non-synonymous mutations in the corresponding residues. *P*-value for each mutation cluster score was computed using the null hypothesis distribution of the mutation score estimated by randomly permuting the mutations within HIF-1α. (**b**,**c**) SET7/9-dependent methylation of HIF-1α (**b**) and protein stability of HIF-1α (**c**) were determined in HeLa cells expressing the indicated HIF-1α MTs. (**d**) *In vitro* methylation assay of HIF-1α WT, K32A and MTs from cancer patients was performed by SET7/9 WT or enzymatic MT (H297A). (**e**) Photomicrographs of Transwell cell migration assays of *Hif1a*^*−/−*^ MEFs reconstituted with the indicated proteins under normoxic conditions for 12 h. Representative images are shown for each group. Bar graph shows the mean number of cells per filter±s.d. (*n*=4 for each group, *t*-test, *P<0.05, **P<0.01). (**f**) In the cytoplasm, HIF-1α protein stability is regulated by PHD-dependent hydroxylation. The hydroxylated HIF-1α binds to VHL for CUL2-dependent degradation by 26S proteasomes under normoxic conditions to maintain low HIF-1α protein levels. In contrast, SET7/9-dependent methylation and LSD1-dependent demethylation of HIF-1α regulate protein stability primarily in the nucleus in a hydroxylation- and VHL-independent manner during normoxia and long-term hypoxia.

We searched for HIF-1α mutations occurring in various human cancers, to identify a potential link between HIF-1α methylation and cancer progression. The catalogue of somatic mutations in cancer and cancer cell line encyclopedia, which are open-access resources for the interactive exploration of multidimensional cancer genomics data sets, were used to identify HIF-1α mutations in human cancers^50^,^51^. Although a mutation of HIF-1α at the K32 site where HIF-1α methylation by SET7/9 occurs was not detected in the database, amino acids such as S28 and R30 near the K32 methylation site were found to be frequently mutated in various human cancers (Fig. 7a and Supplementary Fig. 4). For example, a HIF-1α S28Y mutation was reported in human oesophageal, haematopoietic and lymphoid cancers^51^, and a HIF-1α R30Q mutation was reported in melanoma^51^,^52^.

To examine whether the mutations of HIF-1α occurring in cancer affect its methylation status and lead to the regulation of protein stability, we performed methylation assays with SET7/9 using various HIF-1α MTs in the presence of MG132. HIF-1α methylation was detected from S14R, R17G, R18Q and R19Q mutations as in the case of WT but not from S28Y and R30Q mutations as in the case of the K32A mutation (Fig. 7b). Given that the S28 and R30 sites correspond to SET7/9 target sites in the basic helix-loop-helix domain, we hypothesized that S28Y and R30Q mutations of HIF-1α might have impaired SET7/9-dependent methylation, resulting in increased stability of the HIF-1α MT protein. To examine this possibility, we determined whether SET7/9 modulates HIF-1α MT protein stability. Intriguingly, R17G, R18Q and R19Q mutations of HIF-1α were affected by SET7/9, leading to the destabilization of HIF-1α; however, S28Y and R30Q mutations of HIF-1α within the SET7/9 consensus sequence were resistant to methylation-dependent degradation (Fig. 7c). In parallel, *in vitro* methylation assay confirmed that GST-SET7/9 methylated HIF-1α WT and R17G MT, but failed to methylate K32A and R30Q MT (Fig. 7d).

Furthermore, we performed Transwell cell migration assays to determine the migratory potential of *Hif1a*^*−/−*^ MEFs reconstituted with HIF-1α WT, K32A, R17G or R30Q. SET7/9 expression decreased the migratory potential of HIF-1α WT- or R17G-reconstituted MEFs and LSD1 expression increased the migratory potential of these MEFs. However, SET7/9 and LSD1 expression failed to affect the migratory properties of *Hif1a*^*−/−*^ MEFs reconstituted with HIF-1α K32A or R30Q (Fig. 7e). These data suggest the potential importance of the HIF-1α methylation status within SET7/9 consensus sites in human cancers.

## Discussion

As the function of HIF-1α is regulated primarily at the level of protein stability, most previous studies have focused on the regulation of PHD enzymatic activities resulting in HIF-1α degradation. During hypoxia, the enzymatic activities of PHDs decrease, thus allowing decreased HIF-1α proline hydroxylation. Then, HIF-1α escapes VHL binding and 26S proteasome-dependent degradation. Not only proline hydroxylation but also other posttranslational modifications of HIF-1α are responsible for regulating HIF-1α stability. We found that SET7/9-dependent methylation and LSD1-dependent demethylation of HIF-1α regulate protein stability primarily in the nucleus in a proline hydroxylation- and VHL-independent manner during normoxic and hypoxic conditions (Fig. 7f). We speculate that HIF-1α methylation-dependent degradation may be a fine-tuned process in the nucleus that functions to eliminate both leaky pools of HIF-1α under normoxic conditions and the remaining pool of HIF-1α during long-term hypoxia for the onset of efficient transcriptional activation of HIF-1α.

Among various posttranslational modifications, methylation plays a role in many nuclear processes, such as transcriptional regulation and replication. However, most previous studies have highlighted histone methylation, thus making it difficult to use animal models to address the physiological function of methylation *in vivo*. In the present study, we show that HIF-1α is methylated by SET7/9 and demethylated by LSD1 in the nucleus as in the case of histones. Although we cannot exclude the possibility that other methyltransferases can methylate other lysine sites of HIF-1α depending on different upstream signals, hypoxia-dependent HIF-1α methylation and demethylation at K32 site is conducted by SET7/9 and LSD1, respectively. The finding that HIF-1α methylation affects protein stability led us to generate HIF-1α methylation-deficient mice to explore the biological function of HIF-1α methylation *in vivo*. *Hif1a*^*KA/KA*^ mice exhibited enhanced retinal angiogenesis and tumour vascularization via HIF-1α stabilization, indicating the potential involvement of SET7/9 and LSD1 in regulating retinal and tumour angiogenesis.

*Set7/9* KO mice are normal and largely indistinguishable from their WT littermates in both viability and fertility^53^; thus, the roles of SET7/9 in cancer have not been explored in mouse models. However, several reports have suggested potential roles for SET7/9 in cancer. The SET7/9-mediated methylation of p53 has been shown to facilitate its acetylation, which subsequently increases p53 protein stability^43^,^54^. LSD1 is overexpressed in many cancers and LSD1 inhibition by amine oxidase inhibitors impairs cancer proliferation^55^,^56^,^57^. Furthermore, LSD1 has been reported to promote androgen receptor- and oestrogen receptor-dependent transcription in prostate and breast cancer cells, respectively^35^,^58^,^59^,^60^. Our data showing that LSD1 overexpression in cancer stabilizes HIF-1α and facilitates tumour angiogenesis may explain better how LSD1 promotes not only hormone-dependent cancers but also other types of cancer.

Previously, we reported that methylation-dependent ubiquitination machinery including the DDB1–CUL4-associated factor 1/DDB1/CUL4 E3 ubiquitin ligase complex recognizes monomethylated RORα induced by EZH2 (ref. 30). The oncogenic function of EZH2 may be augmented by the methylation-dependent degradation of tumour suppressive proteins such as RORα in cancer, thus providing an attractive prototype, suggesting the cross-regulation of oncogenes and tumour suppressor genes for efficient tumour progression. The identification of an adaptor molecule containing a methyl recognition domain linking methylated HIF-1α to the E3 ubiquitin ligase complex for degradation would be helpful for understanding methylation-dependent HIF-1α degradation in the nucleus and its biological importance in cancers.

HIF-1α overexpression caused by genetic alteration has been reported in various human cancers^61^,^62^. A remarkable frequency of common genetic alterations that are associated with HIF-1α expression occurs in cancer patients. For example, the loss-of-function of VHL by genetic alteration results in the constitutive expression of HIF-1α and in onset of VHL disease, a dominantly inherited familial cancer syndrome characterized by susceptibility to retinal and central nervous system haemangioblastomas, clear cell renal cell carcinomas, pheochromocytoma, pancreatic islet cell tumours and renal, pancreatic and epididymal cysts^63^,^64^. The loss-of-function of p53 has been shown to increase HIF-1α protein levels and HIF-1α transcription activity in cancers. Although genetic alterations of various genes have been shown to affect HIF-1α expression, genetic mutations of HIF-1α in cancer have not been well studied. In addition, it has been shown that HIF-1α functions as a tumour suppressor in the context of kidney cancer^65^.

Taken together, our studies demonstrate that a methylation/demethylation cycle is involved in the regulation of HIF-1α stability in hypoxia signalling pathways, resulting in enhanced retinal angiogenesis and tumour vascularization *in vivo* in *Hif1a*^*KA/KA*^ mice. Our findings indicate that S28Y and R30Q mutations of HIF-1α within the SET7/9 consensus sequence makes HIF-1α resistant to methylation-dependent degradation. These data suggest the potential importance of the HIF-1α methylation status within SET7/9 consensus sites in human cancers and provide an avenue for the development of future anticancer therapeutics.

## Methods

### Cell culture

MEFs, HEK293T and HeLa cells (ATCC) were cultured in DMEM medium supplemented with 10% fetal bovine serum (Welgene) with penicillin (100 U ml^−1^) and streptomycin (100 μg ml^−1^). The cell lines have been tested for *Mycoplasma* contamination.

### Antibodies

The following commercially available antibodies were used: anti-HIF-1α (NB100–132, Novus; 10006421, 1:1,000 dilution for IB analysis, Cayman; 1:1,000 dilution for IB analysis, 1:200 for IF analysis; MAB 1536, R&D Systems, 1:1,000 dilution for IB analysis); anti-HIF-2α (NB100–122, Novus, 1:1,000 dilution for IB anlysis); anti-Xpress (R910-25, Invitrogen, 1:5,000 dilution for IB analysis); anti-FLAG (F3165, Sigma, 1:10,000 dilution for IB analysis); anti-methyl-Lys (ab23366, Abcam); anti-CD31 (clone 2H8, MAB1398Z, Millipore, 1:200 dilution for immunohistochemical (IHC) analysis); anti-HA (MMS-101R, Covance, 1:5,000 dilution for IB analysis); anti-VEGF (AF493NA, R&D System, 1:200 dilution for IHC analysis); anti-EPO (sc-7956, 1:1,000 for IB analysis), anti-Brn3b (sc-6026, 1:200 dilution for IHC analysis) from Santa Cruz; anti-LSD1 (#2139, 1:1,000 dilution for IB analysis), anti-hydroxyl-HIF-1α (#3434, 1:5,000 dilution for IB analysis), anti-Caspase3 (#9661, 1:200 dilution for IHC analysis), anti-Ki-67 (#9027, 1:100 dilution for IHC analysis) and anti-SET7/9 antibodies (#2813, 1:1,000 dilution for IB analysis) from Cell Signalling. Anti-HIF-1α-K32 methyl antibodies were generated by Abfrontier (South Korea, 1:5,000 dilution for IB analysis).

### Animals

Male C57BL/6J mice at 8–10 weeks of age were used in the experiments. The mice were placed in a hypoxic chamber with a constant flow of 10% oxygen balanced with nitrogen for the indicated times. Food and water were available *ad libitum*. For DMOG treatment, 2 mg of DMOG was dissolved in 0.1 ml PBS and injected intraperitoneally into male mice. All animal procedures were approved by the Institutional Animal Care and Use Committee of Seoul National University.

### Generation of *Hif1a* ^*KA/KA*^ knock-in mice

To replace lysine 32 of HIF-1α with alanine, an NheI site was introduced into the second exon of HIF-1α, into which an flippase recognition target (FRT)-flanked Puro^r^ cassette was inserted. Lysine to alanine substitutions were introduced by site-directed mutagenesis, which generated the AfeI site. A targeting vector containing this alteration was electroporated into embryonic stem cells and positive clones with homologous recombination at the *Hif1a* locus were selected for electroporation with a plasmid expressing protamine-cre recombinase to remove the Puro^r^ cassette. A heterozygous *Hif1a*^*KA/+*^ ES clone was selected and injected into mouse blastocysts, yielding chimeric mice that transmitted the MT allele. The F7 generation was genotyped by PCR analysis of tail DNA samples using allele-specific primers and the MT mice were confirmed by both AfeI digestion and PCR product sequencing. The genotyping primers used were as follows: primer forward: 5′- GTAGGTGGGAAGGTATTGATG -3′ and primer reverse: 5′- AGAACTCACCG GCATCCAGAAG -3′. The full-size image of agarthe gel is shown in Supplementary Fig. 7.

### Quantitative reverse transcriptase–PCR

mRNA abundance was detected using an ABI Prism 7500 system and 2X PreMix SYBR Green (Enzynomics). Primer pairs were designed to amplify 90–200 bp mRNA-specific fragments and were confirmed as unique products by melting curve analysis. The PCR conditions were as follows: 95 °C (15 min) and 40 cycles of 95 °C (30 s), 60 °C (30 s) and 72 °C (30 s). The quantity of mRNA was calculated using the ΔΔCt method and normalized to that of β-actin. All reactions were performed as triplicates. The following primers were used: *Vegf-a* forward: 5′- TGATGGAAGACTAGACAAAGTTCA -3′, *Vegf-a* reverse: 5′- TTTTCCACCAGTTCCA ACTTGA -3′; *Glut1* forward: 5′- AGAGGTGTCACCTACAGCTC -3′, *Glut1* reverse: 5′- AA CAGGATACACTGTAGCAG -3′; *Epo* forward: 5′- gctggcttagccctctcac -3′, *Epo* reverse: 5′- ctgtccgctcctagcatgt -3′; *Lsd1* forward: 5′- CGGCATCTACAAG AGGATAAAACC -3′, *Lsd1* reverse: 5′- CGCCAAGATCAGCTACATAGTTTC -3′; and *Hif-1a* forward: 5′- CAGAGCAGGAAAGAGAGTCATAGAAC -3′, *Hif-1a* reverse: 5′- TTTCGCTT CCTCTGAGCATTC -3′.

### Ubiquitination assay

HeLa cells were transfected with combinations of plasmids including HisMax-ubiquitin. After the cells were incubated for 48 h, they were treated with MG132 (10 μg ml^−1^) for 6 h, lysed in buffer A (6 M guanidium-HCl, 0.1 M Na_2_HPO_4_/NaH_2_PO_4_, 0.01 M Tris-HCl pH 8.0, 5 mM imidazole and 10 mM β-mercaptoethanol) and incubated with Ni^2+^-NTA beads (Qiagen) for 4 h at room temperature. The beads were washed sequentially with buffer A, buffer B (8 M urea, 0.1 M Na_2_PO_4_/NaH_2_PO_4_, 0.01 M Tris-Cl pH 8.0 and 10 mM β-mercaptoethanol) and buffer C (8 M urea, 0.1 M Na_2_PO_4_/NaH_2_PO_4_, 0.01 M Tris-Cl pH 6.3 and 10 mM β-mercaptoethanol). Bound proteins were eluted with buffer D (200 mM imidazole, 0.15 M Tris-Cl pH 6.7, 30% glycerol, 0.72 M β-mercaptoethanol and 5% SDS) and were subjected to IB analysis.

### Soft agar colony formation assay

MEFs were immortalized by 3T3 protocol. The anchorage-independent growth of MEFs was determined by analysing colony formation in soft agar. Cells (10^5^) were placed in DMEM media containing 0.4% noble agar (Sigma, A5431) and 10% fetal bovine serum for 5 weeks in 5% CO_2_ incubator.

### Generation of the OIR mouse model

The OIR mouse model was generated according to previous report^49^. Briefly, newborn mice at P7 and their nursing mothers were exposed to 75% oxygen in a hyperoxic chamber (ProOx Model 110, BioSpherix, NY) for 5 days and then were returned to room air for 5 days. Their retinas were harvested on P17. The mice were handled in accordance with the ARVO Statement for the Use of Animals in Ophthalmic and Vision Research (http://www.arvo.org/about_arvo/policies/statement_for_the_use_of_animals_in_ophthalmic_and_visual_research/).

### Histological analysis of retinal angiogenesis

The retinas were incubated with isolectin B4 (L2140, Sigma) overnight with one or more of the following antibodies: hamster anti-CD31 monoclonal antibody, rabbit anti-HIF-1α polyclonal antibody, goat anti-VEGF polyclonal antibody or goat anti-Brn3b polyclonal antibody^49^. After washing several times, the samples were incubated for 4 h at room temperature with fluorescein isothiocyanate (FITC)-conjugated streptavidin (BD Pharmingen, 1:1,000 dilution) or the following antibodies: FITC-conjugated anti-hamster IgG (Jackson ImmunoResearch, 1:1,000 dilution), Cy3-conjugated anti-goat IgG antibody and Cy3- or Cy5-conjugated anti-rabbit IgG (Jackson ImmunoResearch, 1:1,000 dilution). For the control experiments, the primary antibody was omitted or substituted with pre-immune serum. Whole-mount or sectioned stained retinas were visualized and digital images were obtained using a Zeiss LSM 510 or 780 confocal microscope equipped with argon and helium-neon lasers (Carl Zeiss). Morphometric analyses of the retina were made using ImageJ software (http://rsb.info.nih.gov/ij)^66^ or LSM Image Browser (Carl Zeiss). Radial length of blood vessels in postnatal retina was measured as the shortest distance from the optic nerve head to the peripheral vascular front in each quadrant retina. Vascular density in whole-mounted retina was calculated as CD31^+^ blood vessel area divided by total measured area of the retina and presented as a percentage. Neovascular tuft and avascular areas in OIR retina were measured using the Lasso tool of Adobe Photoshop software as previously described^67^. Signal intensities of HIF1α were measured in the avascular area of the retina and analysed using Image J software.

### Tumour models and histological analysis

Murine LLC cells were purchased from the American Type Culture Collection. To generate tumour models, suspensions of LLC cells (1.5 × 10^6^ cells in 100 μl) were implanted subcutaneously into the dorsal flanks of mice. Tumour volume was measured with a caliper every 2 days. Tumour volume was calculated according to the formula 0.5 × *A* × *B*^2^, where *A* is the greatest diameter of a given tumour and *B* is its perpendicular diameter. At 18 days after tumour implantation, the mice were anaesthetized by intramuscular injection of anaesthetics (ketamine 80 mg kg^−1^ and xylazine 12 mg kg^−1^) and primary tumours were harvested, processed and sectioned for histological analyses. In brief, frozen tumour tissues embedded in OCT freezing medium (Leica) were cut into 50 μm sections and incubated with hamster anti-CD31, anti-Ki67 or anti-caspase3 antibody overnight. After the samples were washed several times, they were incubated for 2 h at room temperature with Cy3- or FITC-conjugated anti-hamster IgG (Jackson ImmunoResearch, 1:1,000 dilution) or Cy3-conjugated anti-rabbit IgG (Jackson ImmunoResearch, 1:1,000 dilution). Next, the samples were mounted and imaged using a LSM510 confocal microscope (Carl Zeiss). Density measurements of blood vessels, hypoxic area, apoptotic area and necrotic areas were performed with Image-J software. Number of Ki67+ cells were manually counted and averaged. To analyse the hypoxia in the tumour, Hypoxyprobe-1 (60 mg kg^−1^, Natural Pharma International) was intravenously injected 60 min before perfusion fixation. Tumours were then collected, processed, sectioned and stained with FITC-conjugated anti-Hypoxyprobe antibody (1:1,000 dilution).

### Xenograft assay

HIF-1α short hairpin RNA stably expressing cells (targeting for 3′-untranslated region: 5′- TATGCACTTTGTCGCTATT AA -3′) were reconstituted with either HIF-1α WT^R^ or K32A^R^ (short hairpin RNA-resistant forms). For tumour formation *in vivo*, cells (10^6^) with equal volume of matrigel (BD Biosciences) were injected subcutaneously at the left flank with HIF-1α WT- and right flank with HIF-1α K32A stably expressing cells into 5-week-old athymic *nu/nu* female mice (*n*=10). Tumours were measured weekly and the experiment was terminated at 4 weeks after injection. Tumours were excised and weighed. Tumour volumes were measured 1/2 × length^2^ × width.

### *In vitro* methylation and demethylation assays

*In vitro* methylation assays were performed by incubating GST-HIF-1α and GST-SET7/9 proteins in methylation buffer (50 mM Tris-HCl pH 8.5, 20 mM KCl, 10 mM MgCl_2_, 10 mM β-mercaptoethanol and 250 mM sucrose) with 1 μCi of ^3^H-SAM at 30 °C overnight. For *in vitro* demethylation assay, methylation of GST-HIF-1α was performed on GST bead-bound HIF-1α proteins for overnight by adding GST-SET7/9 proteins. The beads were extensively washed with wash buffer (50 mM NaH_2_PO_4_ pH 8.0, 10 mM Tris-HCl pH 8.0, 500 mM NaCl and 0.5% Triton X-100) to remove bead-bound SET7/9 protein, followed by addition of His-LSD1 protein in demethylation buffer (50 mM Tris-HCl pH 8.5, 50 mM KCl, 5 mM MgCl_2_, 5% glycerol and 0.5 mM phenylmethylsulphonyl fluoride (PMSF)). After incubating the reaction mixtures at 37 °C for overnight, the reaction buffer was removed and 2 × sample buffer were added. The reaction mixtures were boiled for 10 min, then run by SDS–PAGE and analysed by autoradiography. The full-size images of all autoradiographs and Coomassie stainings are shown in Supplementary Figs 5, 7 and 8.

### Immunoprecipitation assays and immunoblot analysis

For *in vivo* immunoprecipitation experiments, HeLa cells were put in hypoxic chamber for indicated times and treated with 5 μM MG132 (Calbiochem) for 6 h before harvest. Cells were harvested in 1 ml EBC200 buffer (50 mM Tris-HCl pH 8, 200 mM NaCl and 0.5% NP-40) followed by centrifugation for 15 min at 13,000 r.p.m. Nine hundred microlitres of total cell lysates were incubated with indicated antibodies at 4 °C for overnight. Thirty microlitres of a 50% slurry of protein G-Sepharose and A-Sepharose in IP150 buffer (25 mM Tris-HCl pH 7.8, 1 mM EDTA, 10% glycerol, 150 mM NaCl and 0.1% NP-40) were then added to the reaction mixtures and incubated for 2 h at 4 °C. After rapid centrifugation, the resulting Sepharose pellets were washed five times with IP150 buffer and boiled for 7 min with addition of 2 × sample buffer. Co-immunoprecipitated proteins were analysed by SDS–PAGE, followed by immunoblotting using anti-HIF-1α, anti-SET7/9 or anti-LSD1 antibodies (1:1,000) in 3% BSA diluted in PBS-T. After washes in PBS-T, the membrane was incubated for 1 h in the presence of the species-appropriated horseradish peroxidase-conjugated secondary antibody (Jackson) and then washed in PBS-T. Immunolabelled proteins were visualized using LumiFlash Ultima Chemiluminescent substrate (Visual Protein) by LAS-4000 mini (Fuji). The full-size images of all immunoblots are shown in Supplementary Figs 5–8.

### Purification of HIF-1α-binding proteins

HIF-1α-binding proteins were purified from extracts of HEK293T cells expressing Flag-tagged HIF-1α. As a negative control, mock purification from HEK293T cells expressing an empty vector was performed. The HIF-1α-binding proteins were precipitated using Flag M2 agarose beads (Sigma, 100 μl of 50% slurry) from ∼100 mg of cell extracts. After overnight incubation at 4 °C, the beads were washed three times with a BC150 buffer (20 mM Tris-HCl pH 7.9, 15% glycerol, 1 mM EDTA, 1 mM dithiothreitol, 0.2 mM PMSF, 0.05% Nonidet P40 and 150 mM KCl), two times with a BC300 buffer (20 mM Tris-HCl pH 7.9, 15% glycerol, 1 mM EDTA, 1 mM dithiothreitol, 0.2 mM PMSF, 0.05% Nonidet P40 and 300 mM KCl), two times with a BC150 buffer and three times with a TBS buffer (50 mM Tris-HCl pH 7.4 and 150 mM NaCl), to remove nonspecific bindings, and the bound proteins were eluted by competition with the Flag peptide (0.1 mg ml^−1^). The eluted proteins were resolved by SDS–PAGE and prepared for LC-MS/MS analysis.

### Immunofluorescence assays

HeLa cells were cultured in poly-D-lysine-coated coverslip. For IF assay, cells were washed two times with PBS buffer and fixed with 2% formaldehyde for 30 min. Cells were then washed two times with 0.1% Triton X-100 in PBS. For permeabilization, cells were incubated in 0.5% Triton X-100 in PBS for 5 min and washed two times with 0.1% PBS-T. Cells were incubated in blocking solutions (5% BSA in 0.1% PBS-T), to block nonspecific binding of the antibody for 30 min, and incubated in primary antibodies diluted in blocking solution. After four washes with 0.1% PBS-T, cells were incubated in secondary antibodies (Invitrogen, Molecular Probes) and 4,6-diamidino-2-phenylindole. After four washes with 0.1% PBS-T, coverslips were mounted with Vectashield (H-1000) and imaged by microscope (Carl Zeiss).

### Statistical analysis

Data were analysed by Student's *t*-tests for group differences, by one-way analysis of variance for condition (normoxia or hypoxia) differences and group differences separately, and by two-way analysis of variance for condition and group differences together using GraphPad Prism software; *P<0.05, **P<0.01, ***P<0.001.

## Additional information

**How to cite this article:** Kim, Y. *et al*. Methylation-dependent regulation of HIF-1α stability restricts retinal and tumour angiogenesis. *Nat. Commun.* 7:10347 doi: 10.1038/ncomms10347 (2016).

## Supplementary Material

Supplementary Information Supplementary Figures 1-8.

Supplementary Data 1 Peptide sequences of HIF-1a-associated polypeptides by LC-MS/MS analysis.
