# Supplementary material for: Methylation-dependent regulation of HIF-1α stability restricts retinal and tumour angiogenesis
Source: Nat Commun. 2016 Jan 13;7:10347. doi: 10.1038/ncomms10347 (PMC4735525; doi:10.1038/ncomms10347)
Supplement: Supplementary Information — Supplementary Figures 1-8. [file ncomms10347-s1.pdf]

Supplementary Figure 1

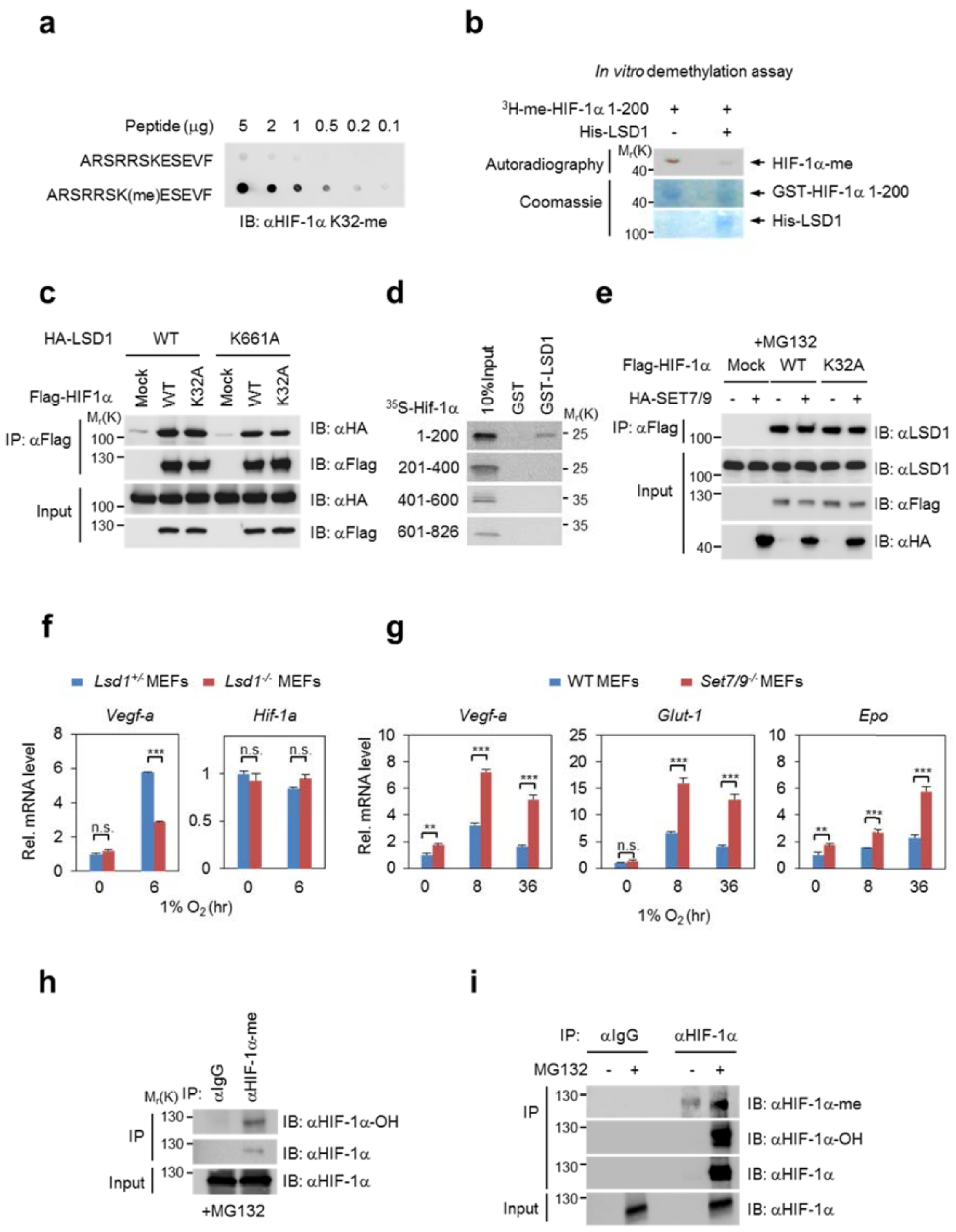

Supplementary Fig. 1. SET7/9-dependent HIF-1α methylation is reversed by LSD1

(**a**) The specificity of antibodies against methylated HIF-1α at the K32 residue (HIF-1α-me) was assessed by dot blot analysis. (**b**) *In vitro* demethylation assays using purified His-LSD1 were performed and HIF-1α methylation was detected by autoradiography. (**c**) Co-immunoprecipitation assay between LSD1 or LSD1 enzymatic mutant (K661A) and HIF1-1α WT or K32A mutant (**d**) GST pulldown assay for binding mapping between HIF-1α and LSD1 (**e**) Immunoprecipitation with anti-Flag antibody from HeLa cells expressing Flag-HIF-1α WT or K32A with or without HA-SET7/9 in the presence of MG132. (**f**) mRNA levels of *Vegf-a*, and *Hif-1a* in *Lsd1*$^{+/-}$ and *Lsd1*$^{-/-}$ MEFs were compared at the indicated times after hypoxic challenge. Data are expressed as mean±s.d. (n=3 for each group, *t*-test, ***p<0.001). (**g**) mRNA levels of *Vegf-a, Glut-1* and *Epo* in WT and *Set7/9*$^{-/-}$ MEFs were compared at the indicated times after hypoxic challenge. Data are expressed as mean±s.d. (n=3 for each group, *t*-test, **p<0.01, ***p<0.001). (**h**) Immunoprecipitation assay with anti-methyl HIF-1α antibody, followed by immunoblot analysis with anti-HIF-1α-OH or anti-HIF-1α antibody was performed in the presence of MG132. (**i**) Immunoprecipitation assay with anti-HIF-1α antibody, followed by immunoblot analysis with anti-methyl HIF-1α or anti-HIF-1α-OH or anti-HIF-1α antibody was performed in the absence or presence of MG132.

Supplementary Figure 2

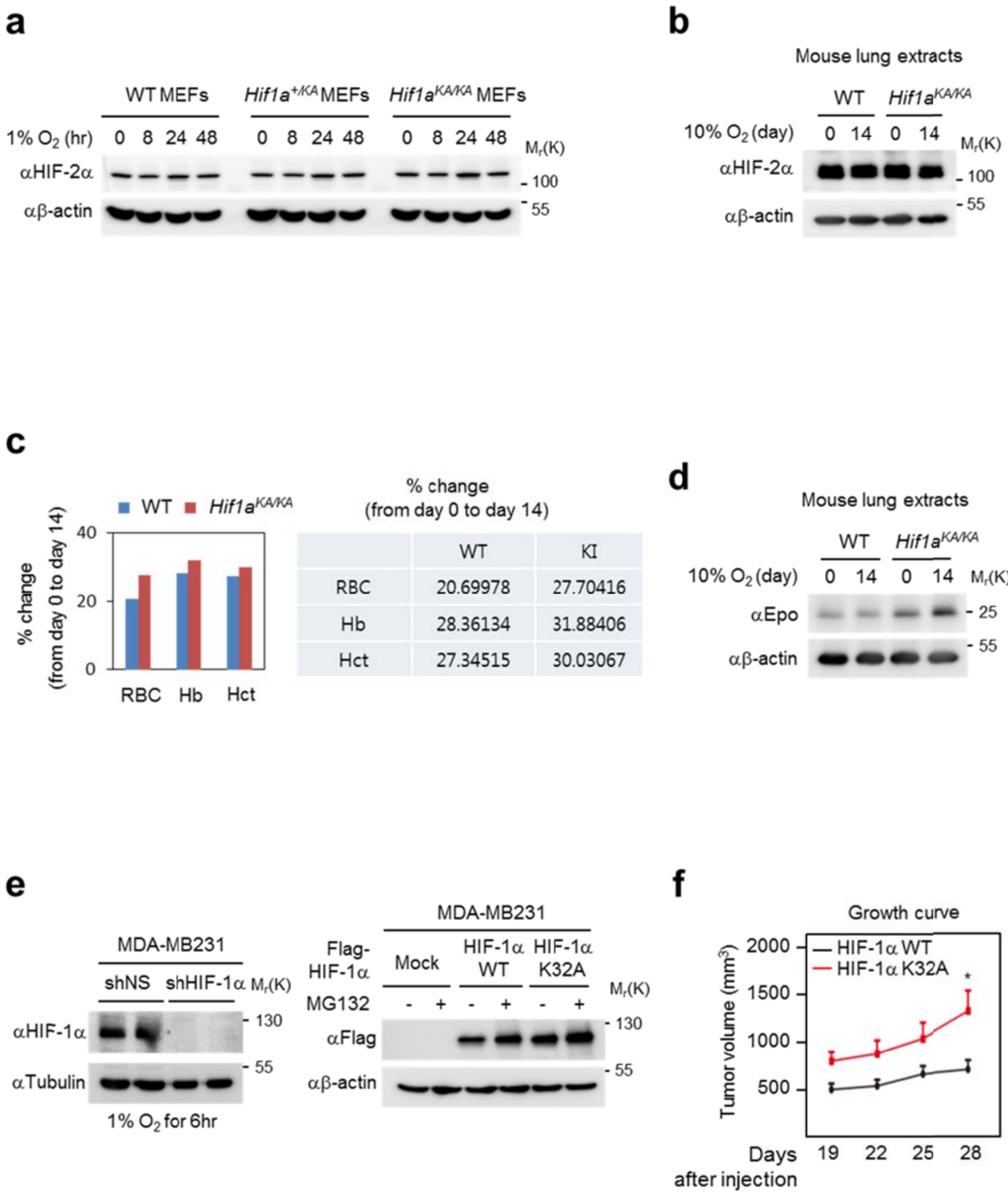

Supplementary Fig. 2. Methylation of HIF-1α does not affect HIF-2α expression

(**a**) HIF-2α protein levels in WT, $Hif1a^{+/KA}$ and $Hif1a^{KA/KA}$ MEFs were compared at the indicated times after hypoxic challenge. (**b**) HIF-2α protein levels in WT and $Hif1a^{KA/KA}$ mouse lung extracts were compared. Mice were incubated in 10% O_2 for indicated times. (**c**) % changes of RBC, haemoglobin and hematocrit in the WT and $Hif1a^{KA/KA}$ mouse were compared. (**d**) Epo protein levels in WT and $Hif1a^{KA/KA}$ mouse lung extracts were compared. Mice were incubated in 10% O_2 for indicated times. (**e**) Immunoblot analysis of MDA-MB231 cells stably expressing control shRNA, HIF-1α shRNA, or HIF-1α shRNA with reconstituted shRNA resistant form of HIF-1α WT or K32A was performed. HIF-1α protein levels were monitored. (**f**) Tumour growth curve. Measurement of tumour volumes in every 3 days starting when the mean tumour size reached 500 mm^3 (day 19). Data are presented as mean+s.e.m. (n=10, two-way ANOVA, *$p<0.05$).

Supplementary Figure 3

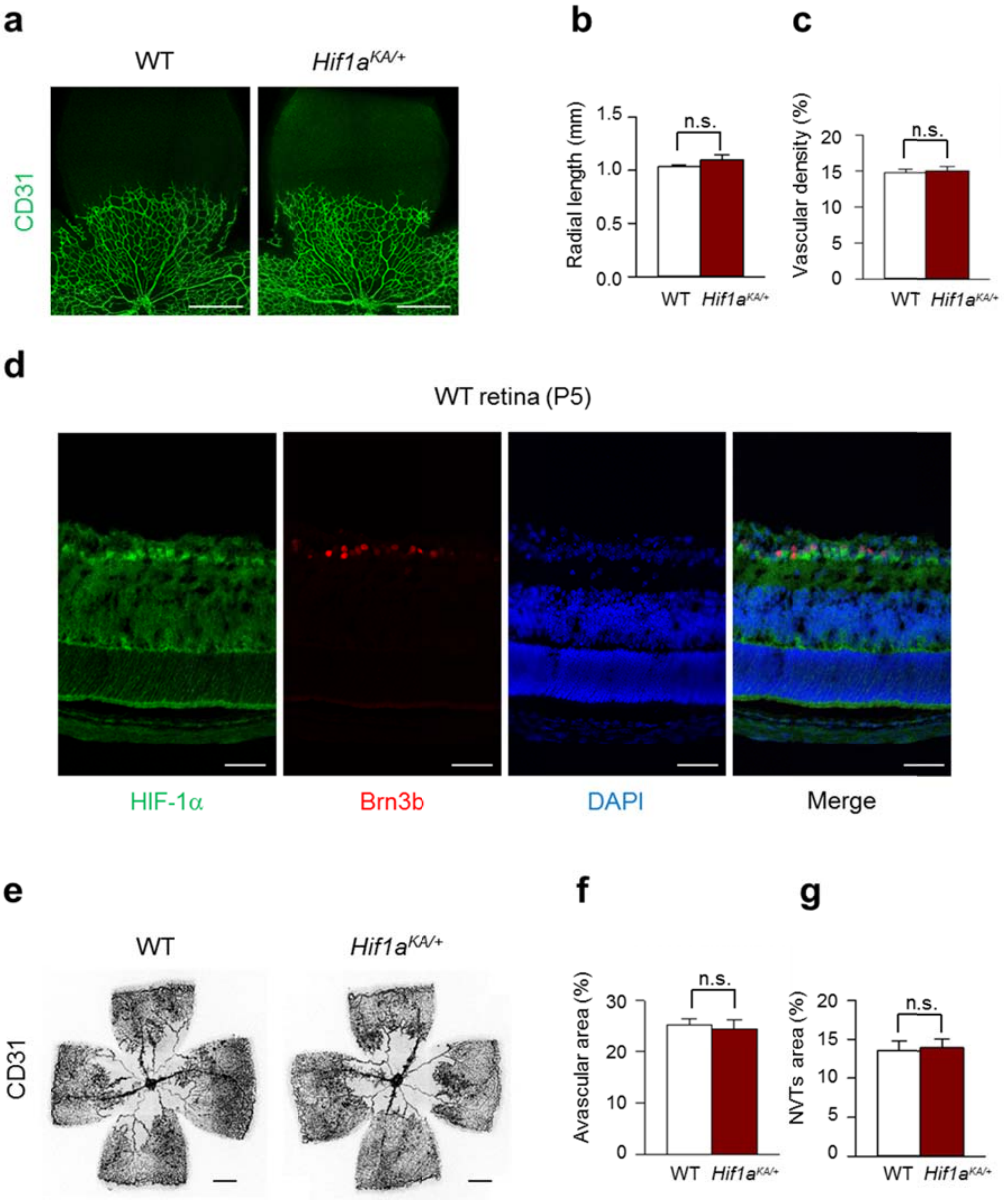

Supplementary Fig. 3. Comparison of retinal angiogenesis in WT and *Hif1a*$^{KA/+}$ mice

(**a-c**) Physiologic retinal angiogenesis at postanatal day 5 (P5) in WT and *Hif1a*$^{KA/+}$mice. (**a**) P5 retina whole mount-stained with CD31. Scale bars, 500 µm. (**b, c**) Comparisons of blood vessel radial length, vascular density (n=4 for each group, *t*-test). (**d**) HIF-1α is most highly expressed in the Brn3b expressing retinal ganglion cell layer. Scale bars, 50 µm. (**e-g**) Pathologic retinal angiogenesis in oxygen-induced retinopathy (OIR) model from WT and *Hif1a*$^{KA/+}$ mice. (**e**) P17 OIR retina wholemount-stained with CD31.Scale bars, 500 µm. (**f, g**) Comparisons of avascular and neovascular tufts (NVT) area (n=4 for each group, *t*-test).

Supplementary Figure 4

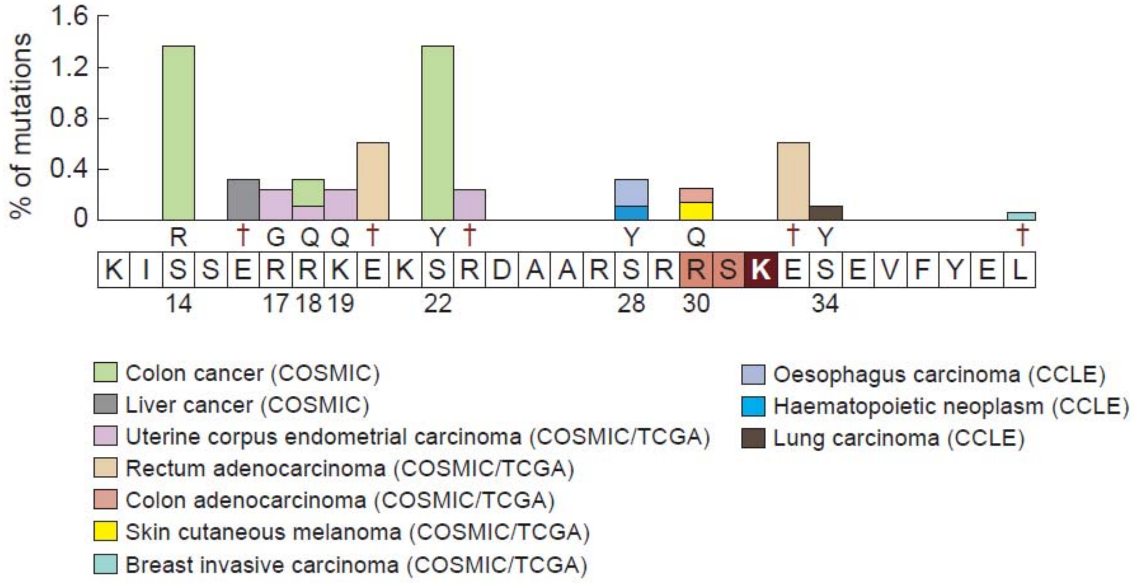

Supplementary Fig. 4. Percentage of tumours for each somatic mutation in the region including two mutation cluster regions near K32 of HIF-1α

Reference sequences for 12 to 40th amino acid residues of HIF-1α are denoted in the boxes. Sequence changes resulted from the mutations are shown above the reference sequences, and synonymous mutations are denoted by the cross (†) symbols. Numbers under the sequence boxes indicate the presence of non-synonymous mutations in the corresponding residues. Tumour percentages (% of mutations) were shown in y-axis for the amino acid residues of HIF-1α.

Supplementary Figure 5

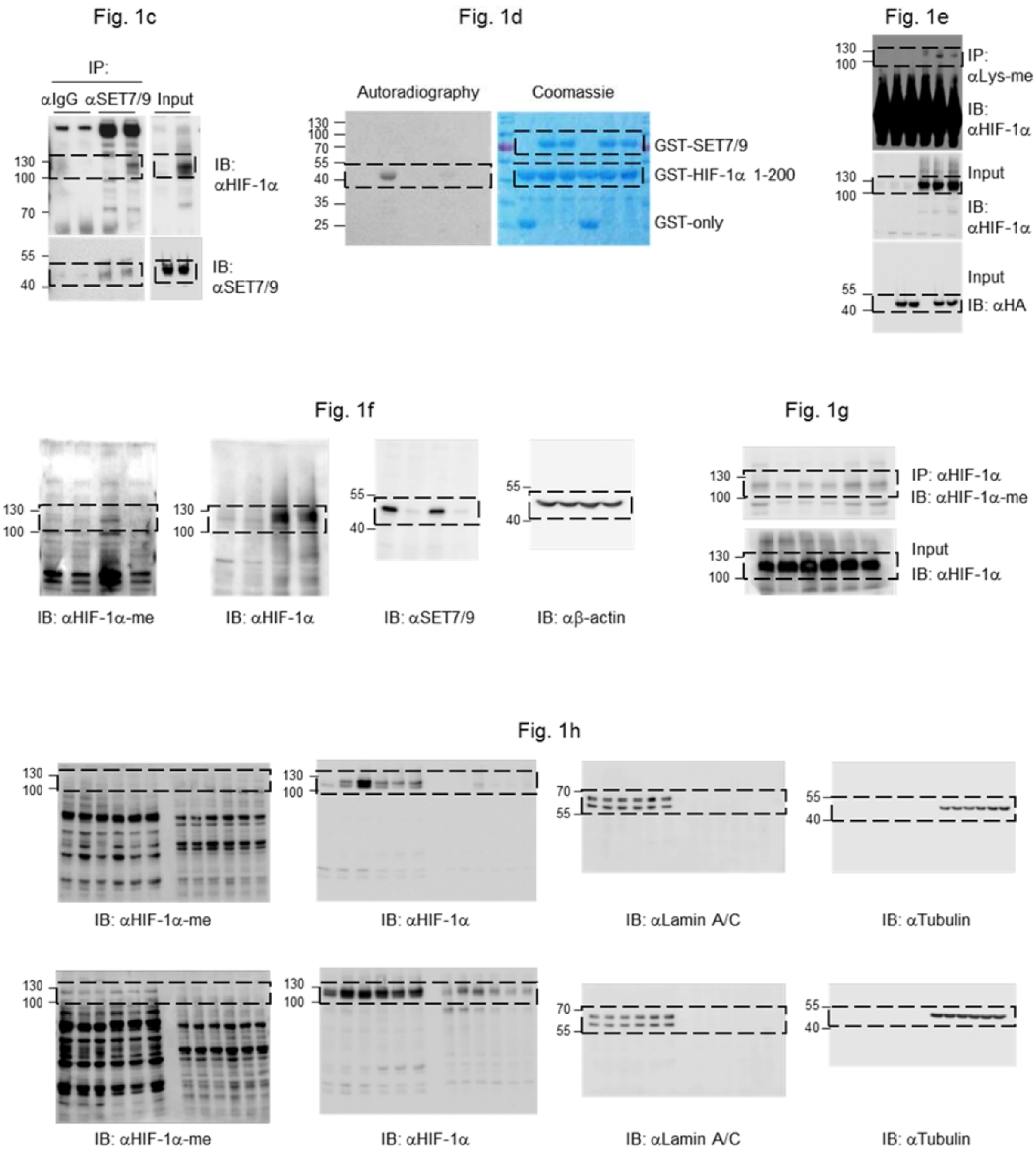

Supplementary Fig. 5. The original full immunoblot, autoradiography or coomassie stained gel images utilized in Figure 1

Supplementary Figure 6

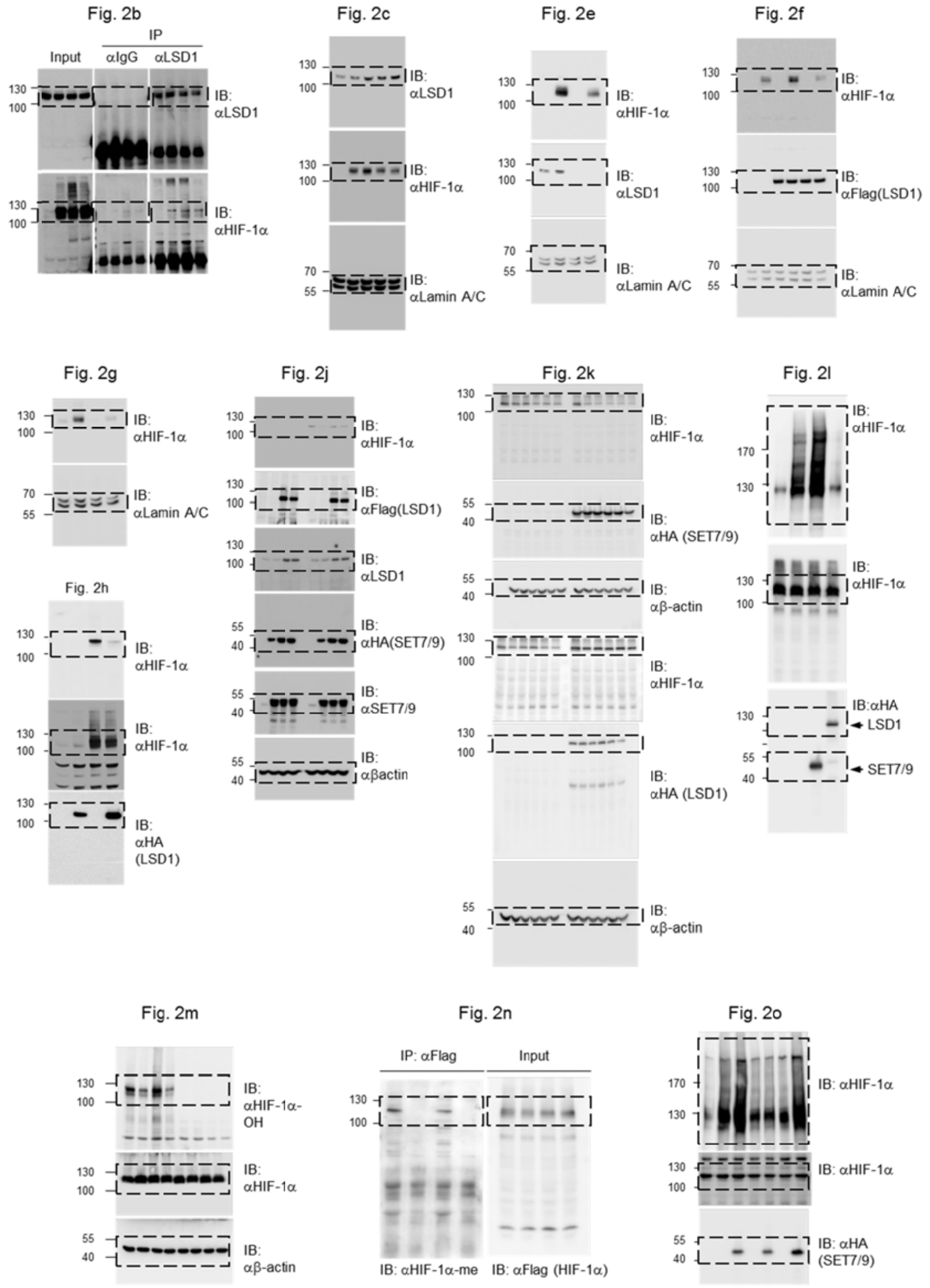

Supplementary Fig. 6. The original full immunoblot images utilized in Figure 2

Supplementary Figure 7

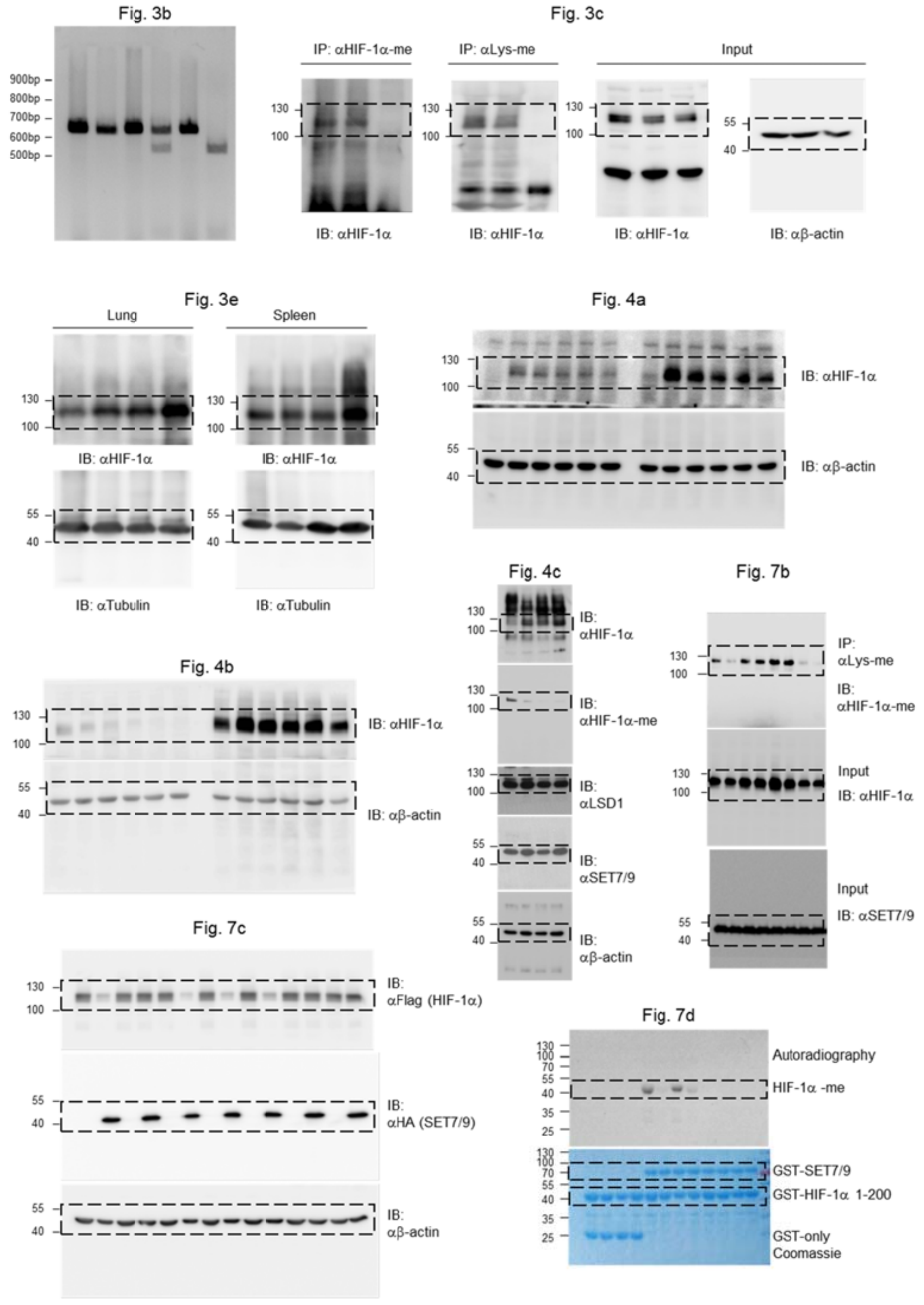

Supplementary Fig. 7. The original full immunoblot, agar gel, autoradiography or coomassie stained gel images utilized in Figures 3, 4, and 7

Supplementary Figure 8

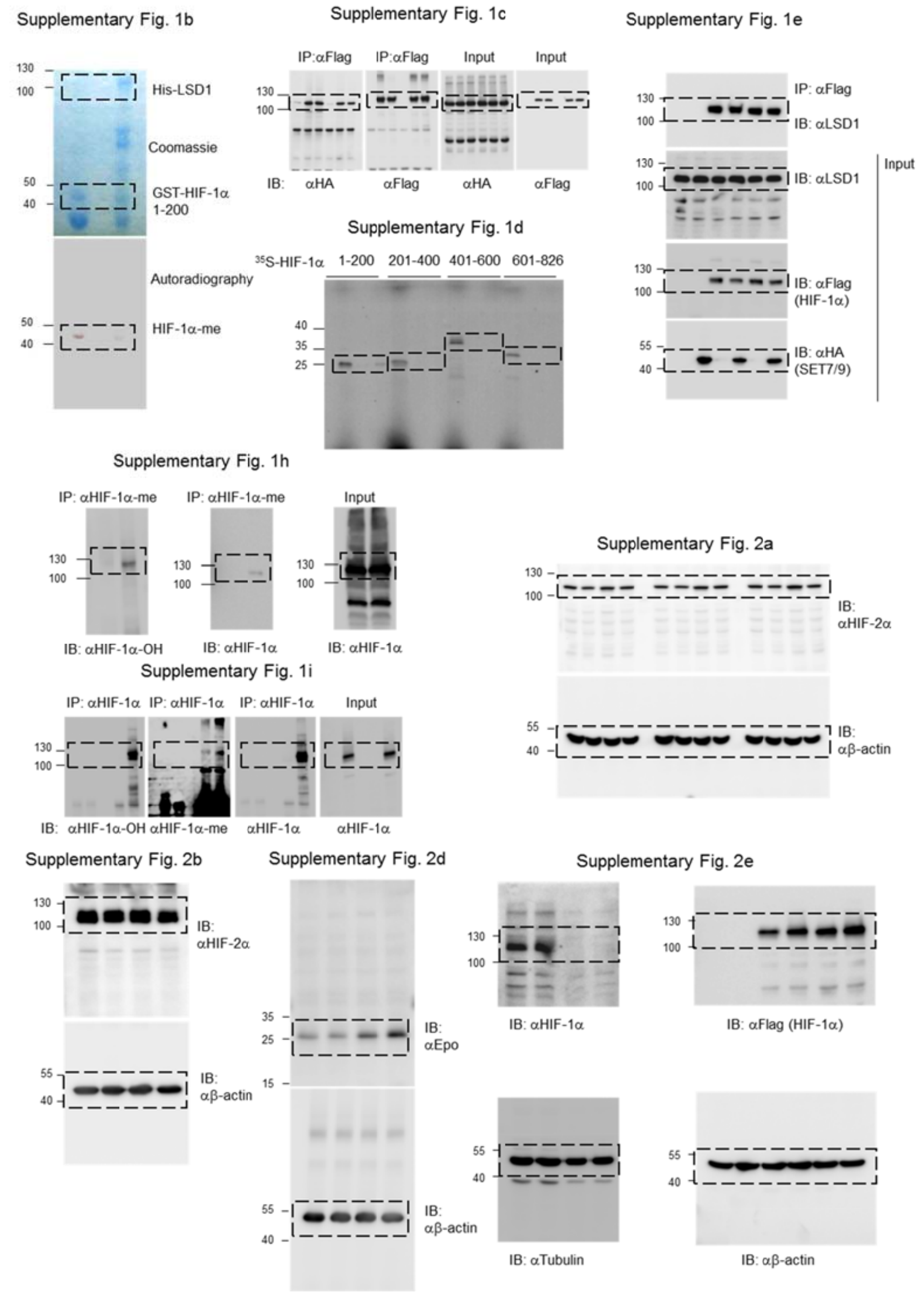

Supplementary Fig. 8. The original full immunoblot, autoradiography, or coomassie stained gel images utilized in Supplementary Figure 1, 2
